# Multimodal neuroimaging fusion in presurgical evaluation of drug-resistant epilepsy: current evidence, clinical integration, and future directions

**DOI:** 10.3389/fnins.2026.1855648

**Published:** 2026-09-03

**Authors:** Yuying Zhao, Mingli Wang, Bo Shen, Wei Luo

**Affiliations:** Department of Radiology, Huzhou Central Hospital, Huzhou, Zhejiang, China

**Keywords:** drug-resistant epilepsy, epileptogenic zone, image fusion, machine learning, multimodal neuroimaging, network neuroscience, presurgical evaluation, surgical outcome

## Abstract

Approximately 30% of patients with epilepsy are drug-resistant, and for many of them surgical resection of the epileptogenic zone (EZ) offers the best chance of seizure freedom. Because the EZ cannot be measured directly by any single test, it must be inferred from the convergence of complementary imaging markers, and multimodal neuroimaging fusion has therefore become central to modern presurgical evaluation. This critical narrative review examines what each modality—structural MRI, [18F]FDG-PET, ictal SPECT/SISCOM, magnetoencephalography and electric source imaging, functional MRI, diffusion tensor imaging, and arterial spin labeling—contributes as a surrogate marker of the epileptic network, and how these are combined through co-registration, three-dimensional integration, hybrid PET/MRI, quantitative methods, and machine learning. Rather than cataloging modalities, we organize the clinical evidence by clinical scenario—including MRI-negative and extratemporal epilepsy, suspected focal cortical dysplasia, discordant non-invasive data, and stereo-EEG planning—and provide a practical framework matching modality combinations to specific clinical decisions. We argue that the central challenge of the field is not the proliferation of techniques but clinical translation: improved localization confidence does not automatically improve seizure freedom, and reported performance figures—whether for multimodal concordance or for artificial-intelligence-based lesion detection—derive largely from retrospective, single-center, or internally validated cohorts and are best interpreted as upper bounds rather than as accuracy deployable across centers. Network-based approaches and personalized computational models, including the Virtual Epileptic Patient, are conceptually powerful but remain largely investigational, and we conclude that multimodal imaging augments rather than replaces expert multidisciplinary evaluation. Prospective multicenter validation and workflow standardization remain the key priorities for clinical translation.

## 1 Introduction

Epilepsy remains one of the most prevalent chronic neurological disorders, affecting approximately 50 million individuals worldwide (Fiest et al., 2017). Despite the expanding armamentarium of antiseizure medications (ASMs), a substantial proportion of patients fail to achieve sustained seizure freedom. Drug-resistant epilepsy (DRE), as formally defined by the International League Against Epilepsy (ILAE), refers to the failure of adequate trials of two tolerated, appropriately chosen, and properly used ASM schedules—whether as monotherapy or in combination—to achieve sustained seizure freedom (Kwan et al., 2010). Epidemiological data consistently indicate that DRE affects approximately 30% of all epilepsy patients, although prevalence estimates vary between 15 and 36% depending on the study setting and population characteristics (Kalilani et al., 2018; Sultana et al., 2021). For these individuals, uncontrolled seizures impose a profound burden encompassing cognitive decline, psychiatric comorbidities, diminished quality of life, and elevated mortality risk including sudden unexpected death in epilepsy (SUDEP) (Perucca et al., 2023).

Surgical resection of the epileptogenic zone (EZ) has been established as the most effective therapeutic strategy for appropriately selected patients with DRE, with long-term seizure freedom rates of 60%−80% in temporal lobe epilepsy and 40%−60% in extratemporal cases (Engel, 2012; Wiebe et al., 2001). Nevertheless, the success of epilepsy surgery hinges critically upon the precise identification and complete resection of the EZ. As articulated in the seminal framework by Rosenow and Lüders (2001), the EZ is defined as “the minimum amount of cortex that must be resected (inactivated or completely disconnected) to produce seizure freedom.” Importantly, the EZ itself is a theoretical construct that cannot be directly measured by any single diagnostic investigation. Rather, its localization must be inferred through the convergence of information derived from multiple surrogate cortical zones: the symptomatogenic zone, the irritative zone, the seizure onset zone (SOZ), the epileptogenic lesion, and the functional deficit zone. Each of these zones is delineated by distinct diagnostic modalities, and the degree of concordance among them ultimately determines the confidence with which a surgical plan can be formulated (Jehi, 2018). This distinction has a direct consequence that frames the present review: because the EZ cannot be measured directly, every neuroimaging modality yields only a surrogate marker of the underlying epileptic network, and the central value of multimodal fusion lies not in the standalone accuracy of any single technique but in triangulating this inherently unmeasurable target through their concordance.

Structural magnetic resonance imaging (MRI) has long served as the cornerstone of presurgical neuroimaging, offering unparalleled anatomical detail for the detection of epileptogenic lesions such as hippocampal sclerosis (HS), focal cortical dysplasia (FCD), and tumors. The ILAE Neuroimaging Task Force has recommended a standardized acquisition protocol—the HARNESS-MRI protocol—incorporating three-dimensional T1-weighted, FLAIR, and high-resolution T2-weighted sequences to optimize lesion detection (Bernasconi et al., 2019). Despite these advances, conventional MRI fails to identify a structural abnormality in 20%−40% of patients with DRE, a condition commonly referred to as “MRI-negative” or “non-lesional” epilepsy (Wang et al., 2020; Muhlhofer et al., 2017). This population poses a particularly formidable clinical challenge, as the absence of a visible lesion is consistently associated with lower rates of seizure freedom following surgery compared with lesional cases (Téllez-Zenteno et al., 2010).

The limitations of relying on a single imaging modality have spurred growing interest in the integration of complementary neuroimaging techniques. Each modality captures distinct yet partially overlapping aspects of brain pathophysiology: [18F]fluorodeoxyglucose positron emission tomography (FDG-PET) reveals interictal hypometabolism, reflecting regions of neuronal dysfunction that frequently extend beyond the structural lesion (Burneo et al., 2015); ictal single-photon emission computed tomography (SPECT), particularly when processed using subtraction ictal SPECT co-registered to MRI (SISCOM), delineates zones of ictal hyperperfusion [as defined by O'Brien et al. (1998)]; magnetoencephalography (MEG) provides millisecond-level temporal resolution for electromagnetic source imaging of interictal epileptiform discharges (Bagić et al., 2011); functional MRI (fMRI) maps both eloquent cortex localization and resting-state network alterations; and diffusion tensor imaging (DTI) reveals white matter microstructural abnormalities and connectivity changes associated with the epileptic network (Zhang et al., 2020). While each of these modalities offers valuable localizing information independently, no single technique possesses sufficient sensitivity and specificity to reliably delineate the EZ in all clinical scenarios.

The concept of multimodal neuroimaging fusion—the systematic integration of structural, functional, metabolic, and electrophysiological imaging data—has thus emerged as a promising paradigm to enhance the precision of presurgical EZ localization. Rather than restating that no single modality is sufficient, we take that premise as our point of departure and ask a more clinically pointed question: how do specific modality combinations improve concordance with invasive intracranial EEG, and when does that improved concordance actually translate into better surgical outcomes (Biagioli et al., 2025)? The clinical rationale for multimodal integration is particularly compelling in MRI-negative cases, where the convergence of PET hypometabolism, SPECT hyperperfusion, and MEG source clusters can collectively define a target region that would otherwise remain elusive on structural imaging alone. Recent investigations have demonstrated that the combination of PET with MRI post-processing techniques such as morphometric analysis programs (MAP) and composite parametric maps (CPM) achieves substantially higher sensitivity (up to 97%) compared with either modality in isolation. Moreover, the advent of hybrid PET/MRI scanners has enabled simultaneous acquisition with precise spatial co-registration, further enhancing diagnostic accuracy (Mouthaan et al., 2019).

Concurrent with these hardware and methodological advances, a paradigm shift has been occurring in the conceptualization of epilepsy pathophysiology—from a focus-centric model to a network-based framework. It is important to characterize the earlier framework accurately: the classical model of Rosenow and Lüders is not defined by the presence of a lesion but by the distinction of cortical zones with different mechanistic roles—the seizure onset, irritative, epileptogenic, symptomatogenic, and functional deficit zones, together with the lesion where one is present. It applies equally to non-lesional cases, and even when a lesion is visible on MRI, imaging alone never establishes that it is epileptogenic; electrophysiological confirmation is essentially always required. The shift we describe is therefore not from lesion-based to network-based evaluation, but from a zone-based conception of a circumscribed epileptogenic region to a distributed-network conception. Accumulating evidence from structural and functional connectomics indicates that DRE involves widespread alterations in brain network topology that extend beyond any single circumscribed zone (Bernhardt et al., 2016). This network perspective has catalyzed the development of multimodal fusion approaches that integrate graph-theoretical analyses of structural connectivity (derived from DTI), functional connectivity (from resting-state fMRI or EEG), and metabolic information (from PET) to characterize the epileptic network architecture and identify nodes critical for seizure generation and propagation. Furthermore, the application of machine learning and deep learning algorithms to multimodal neuroimaging data has shown promising results in automated EZ detection, seizure outcome prediction, and data-driven classification of epilepsy subtypes, heralding a new era of artificial intelligence (AI)-assisted presurgical evaluation.

Despite the rapid proliferation of multimodal fusion methodologies and their increasingly demonstrated clinical utility, several challenges persist. These include the absence of standardized acquisition and processing protocols across epilepsy centers, limited external validation through multicenter studies, and the computational complexity associated with integrating high-dimensional heterogeneous data. Moreover, a critical question is too often left implicit: improved localization confidence does not automatically translate into improved seizure freedom. Distinguishing genuine gains in surgical outcome from gains in diagnostic confidence alone—and identifying where prospective validation remains absent—is, we argue, the central unsolved problem of the field and the organizing question of this review.

**Figure 1:**
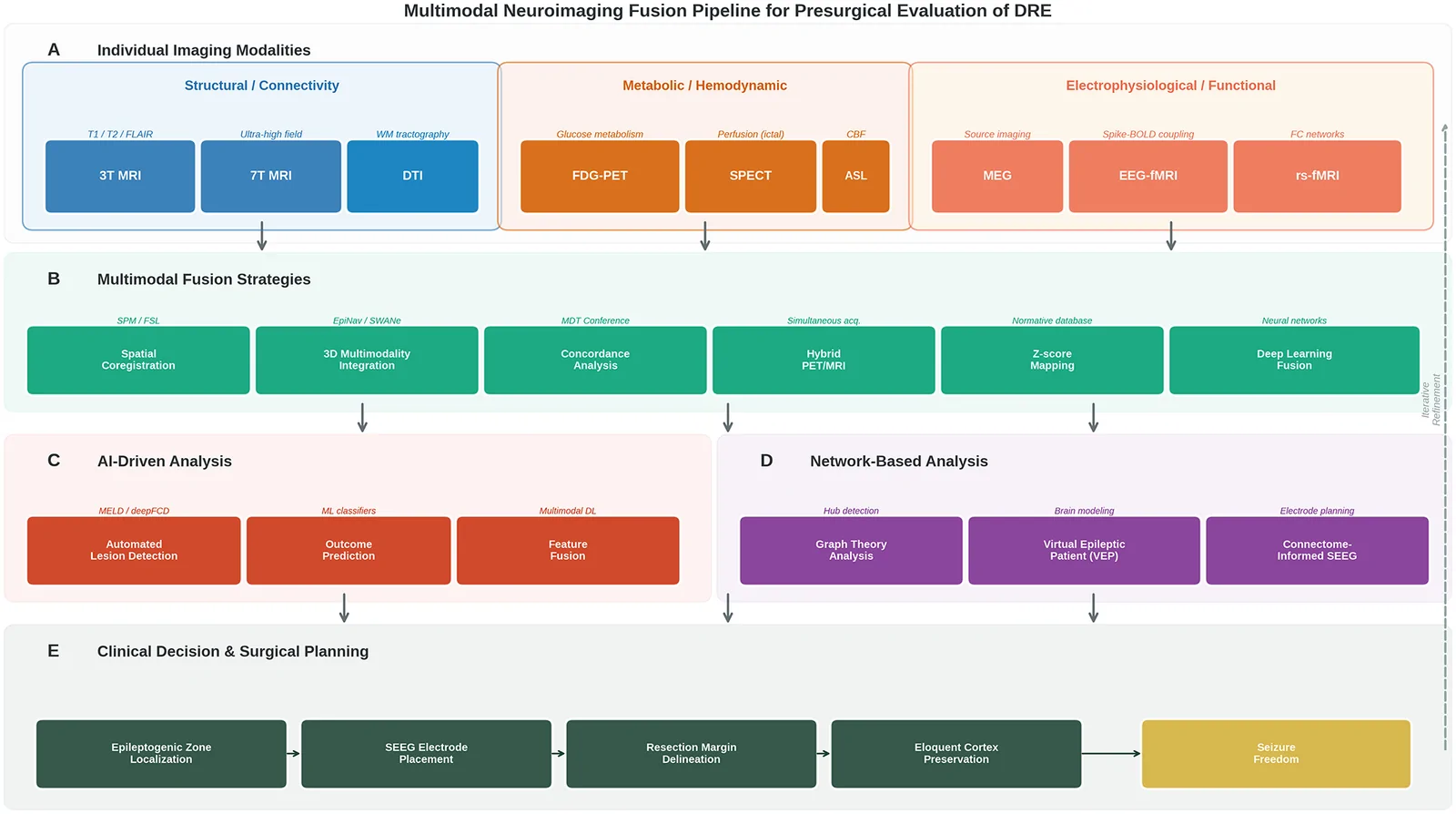
Multimodal neuroimaging fusion pipeline for presurgical evaluation of DRE. The flowchart illustrates the integration process from **(A)** individual structural, metabolic, and electrophysiological imaging modalities, through **(B)** technical fusion strategies, to advanced computational approaches including **(C)** AI-driven analysis and **(D)** network-based analysis. These convergent data streams ultimately guide **(E)** clinical decision-making and surgical planning in an iterative refinement process.

This review is therefore framed around a single organizing argument: because the epileptogenic zone cannot be directly measured, imaging modalities yield only surrogate markers of the epileptic network, and the clinical value of fusion lies in matching specific modality combinations to specific clinical decisions—lesion detection, lateralization, stereo-EEG (SEEG) planning, functional-risk mapping, and outcome prediction—rather than in the accuracy of any single modality in isolation. This is a critical narrative review, and its review strategy is described in Section 2. We then survey what each individual modality contributes as a surrogate marker (Section 3) and examine the technical methods of fusion, from co-registration to artificial-intelligence integration (Section 4). Departing from the conventional modality-by-modality structure, we synthesize the clinical evidence organized by clinical scenario (Section 5)—for example, MRI-negative extratemporal epilepsy, suspected focal cortical dysplasia, discordant non-invasive data, and SEEG planning. We next position network-based approaches as an additional layer of information to be integrated rather than a competing paradigm (Section 6), and critically appraise artificial-intelligence and automated-detection methods with explicit attention to their current limitations (Section 7). Finally, we address the challenges, barriers to clinical translation, and future directions of the field (Section 8), before concluding (Section 9). Throughout, each modality and method closes with a short clinical bottom line summarizing what it adds and its current level of maturity. Figure 1 provides a schematic overview of the multimodal fusion pipeline.

## 2 Review strategy

This article is a critical narrative review rather than a systematic review or meta-analysis. Given the breadth of the field—spanning structural, metabolic, electrophysiological, perfusion, and network-level imaging alongside a rapidly expanding computational literature—our aim was to synthesize and critically appraise the evidence base rather than to exhaustively enumerate all published studies or to derive pooled quantitative estimates. We therefore did not apply a formal PRISMA protocol, and the selection of studies necessarily reflects expert judgment regarding clinical relevance and methodological quality. This framing is made explicit so that the quantitative estimates cited throughout are read as illustrative of the current evidence rather than as the product of a systematic evidence-synthesis procedure.

To identify relevant literature, we searched PubMed/MEDLINE, Embase, and Web of Science for articles published from database inception through 2025, using combinations of the terms “drug-resistant epilepsy,” “refractory epilepsy,” “epilepsy surgery,” “presurgical evaluation,” “epileptogenic zone,” “multimodal,” “image fusion,” “co-registration,” “PET/MRI,” “SISCOM,” “magnetoencephalography,” “magnetic source imaging,” “electric source imaging,” “functional MRI,” “diffusion tensor imaging,” “arterial spin labeling,” “network,” “connectomics,” and “machine learning.” Reference lists of key articles and recent reviews were hand-searched for additional sources. We prioritized peer-reviewed studies that reported clinical validation against intracranial EEG, the resection zone, histopathology, or surgical outcome, and we sought to balance landmark studies that shaped the field with the most recent evidence. Where quantitative estimates are cited, we indicate the study type (single-center series, multicenter study, or meta-analysis) and, where relevant, the reference standard used, so that readers can weigh the strength of the underlying evidence.

## 3 Overview of individual neuroimaging modalities in presurgical evaluation

Before examining how multimodal integration enhances presurgical evaluation, it is essential to understand the individual strengths and inherent limitations of each neuroimaging technique. Each modality interrogates a distinct aspect of brain pathophysiology—structural anatomy, metabolism, perfusion, electrophysiology, or connectivity—and contributes uniquely to the delineation of the cortical zones described by Rosenow and Lüders (2001). The following subsections provide a concise overview of the principal modalities currently employed, with emphasis on their clinical utility and diagnostic performance in the context of drug-resistant epilepsy (DRE). A comparative summary is presented in Table 1.

**Table 1:** Comparative overview of neuroimaging modalities in presurgical evaluation of drug-resistant epilepsy.

Modality	Principle	Spatial resolution	Temporal resolution	Primary clinical value	Sensitivity (TLE/ETLE)	Key limitations
Structural MRI (3T)	Tissue contrast (T1, T2, FLAIR)	Sub-millimeter (~0.5–1 mm)	N/A (static)	Epileptogenic lesion detection; anatomical reference	60%−85%/40%−60% (lesion detection)	20%−40% non-lesional; limited for subtle FCD type I
7T MRI	Ultra-high-field tissue contrast	~0.3–0.5 mm	N/A (static)	Detection of occult lesions in MRI-negative cases	Additional 20%−30% yield over 3T	Limited availability; motion artifacts; SAR constraints
FDG-PET	Interictal glucose hypometabolism	4–6 mm	Minutes (tracer uptake)	Functional deficit zone; lateralization	60%−90%/30%−67%	Hypometabolism exceeds EZ; poor surgical margin delineation
Ictal SPECT/SISCOM	Ictal cerebral hyperperfusion	8–12 mm (SPECT); improved with SISCOM	Seconds (tracer injection)	Seizure onset zone mapping	90%−97%/50%−70% (ictal); SISCOM positive rate ~86%	Logistically demanding; injection timing critical; propagation artifacts
MEG/MSI	Magnetic fields from neuronal currents	2–5 mm (source modeling)	Milliseconds	IED source localization; neocortical focus mapping	82% sensitivity (95% CI 75%−88%); 53% specificity (95% CI 37%−68%) [meta-analysis (Mouthaan et al., 2019)]	Limited for deep/mesial sources; high cost; short recording windows
Electric source imaging (ESI)	Scalp electric potentials from neuronal currents; source reconstruction on individual MRI	5–10 mm (source-model dependent)	Milliseconds	IED/ictal source localization; complements or substitutes for MEG	Comparable to MEG when high-density EEG and individualized head models are used; density-dependent	Volume-conduction and skull effects; accuracy depends on electrode density and head model
EEG-fMRI	Spike-triggered BOLD response	2–3 mm (fMRI)	Seconds (hemodynamic)	Irritative zone mapping; network characterization	Variable (dependent on IED frequency)	Technically demanding; requires sufficient IEDs during scan
Task-based fMRI	BOLD signal during cognitive tasks	2–3 mm	Seconds	Eloquent cortex lateralization (language, memory)	85%−95% concordance with Wada	Limited coverage; task compliance issues in children
rs-fMRI	Spontaneous BOLD fluctuations	2–3 mm	Seconds	Functional connectivity; network lateralization	~70% sensitivity, ~72% specificity (ICA-based)	Susceptible to motion artifacts; limited standardization
DTI	White matter diffusion anisotropy	2–3 mm	N/A (static)	Eloquent tract mapping; perilesional WM integrity	N/A (primarily qualitative/adjunctive)	Crossing fibers; partial volume; limited specificity for EZ
ASL	Endogenous perfusion labeling	3–5 mm	Seconds–minutes	Non-invasive CBF mapping; complement to PET	Moderate (κ~0.70 with PET in TLE)	Lower SNR than PET/SPECT; limited sensitivity for ETLE

ASL, arterial spin labeling; BOLD, blood-oxygen-level-dependent; CBF, cerebral blood flow; DTI, diffusion tensor imaging; ETLE, extratemporal lobe epilepsy; EZ, epileptogenic zone; FCD, focal cortical dysplasia; FDG-PET, [18F]fluorodeoxyglucose positron emission tomography; fMRI, functional magnetic resonance imaging; ICA, independent component analysis; IED, interictal epileptiform discharge; MEG, magnetoencephalography; MSI, magnetic source imaging; rs-fMRI, resting-state functional MRI; SAR, specific absorption rate; SISCOM, subtraction ictal SPECT co-registered to MRI; SNR, signal-to-noise ratio; SPECT, single-photon emission computed tomography; TLE, temporal lobe epilepsy; WM, white matter.

### 3.1 Structural MRI

Structural MRI remains the indispensable first-line imaging investigation in presurgical evaluation, serving as the primary tool for identifying the epileptogenic lesion. High-resolution protocols incorporating three-dimensional T1-weighted (MPRAGE), FLAIR, and hippocampal-oriented T2-weighted sequences—as recommended by the ILAE HARNESS-MRI initiative—have significantly improved the detection of common epileptogenic substrates including hippocampal sclerosis, focal cortical dysplasia (FCD), tumors, and vascular malformations (Bernasconi et al., 2019). The identification of a concordant structural lesion on MRI is arguably the single strongest predictor of favorable surgical outcomes, with seizure freedom rates exceeding 70% in lesional temporal lobe epilepsy compared with 30%−50% in non-lesional cases (Téllez-Zenteno et al., 2010; Noe et al., 2013).

Despite its pivotal role, conventional MRI at 1.5T or even 3T fails to detect a structural abnormality in an estimated 20%−40% of patients undergoing presurgical evaluation—a proportion that is particularly high in extratemporal neocortical epilepsy and in FCD type I (Muhlhofer et al., 2017). Quantitative MRI post-processing techniques have emerged to address this gap. Morphometric analysis programs (MAP) and voxel-based morphometry (VBM) detect subtle cortical thickening and blurring of the gray-white matter junction, while composite parametric maps (CPM) integrate multiple morphometric features into a single statistical map (Hong et al., 2014; Wagner et al., 2011). Furthermore, ultra-high-field 7T MRI has demonstrated incremental diagnostic yield over 3T, revealing previously occult lesions in approximately 20%−30% of MRI-negative cases by exploiting superior spatial resolution and enhanced tissue contrast (Wang et al., 2020). Non-etheless, the accessibility of 7T scanners remains limited to specialized academic centers, constraining its widespread clinical adoption.

***Clinical bottom line:*** *high-resolution structural MRI is the indispensable first-line modality and the anatomical substrate onto which all other data are fused; a lesion identified with the HARNESS protocol remains the single strongest predictor of favorable surgical outcome. Its principal limitation is the 20%*−*40% of patients who remain MRI-negative, which defines the very problem that multimodal fusion is intended to solve. Maturity: routine clinical use*.

### 3.2 [18F]FDG-PET

Interictal [18F]fluorodeoxyglucose positron emission tomography (FDG-PET) is the most widely utilized metabolic imaging modality in epilepsy presurgical assessment, capitalizing on the well-characterized phenomenon of interictal glucose hypometabolism within and around the epileptogenic zone. The zone of hypometabolism typically encompasses the seizure onset zone and the functional deficit zone, although it frequently extends beyond the boundaries of the eventual surgical resection, reflecting widespread network dysfunction rather than a discrete epileptogenic focus (Burneo et al., 2015).

In temporal lobe epilepsy (TLE), FDG-PET demonstrates consistently high sensitivity for lateralization and localization, with reported values ranging from 60 to 90% depending on the reference standard and patient selection (Drzezga et al., 1999). Its performance in extratemporal lobe epilepsy (ETLE), however, is substantially lower, with sensitivity estimates between 30 and 67%, owing to the smaller size and variable depth of extratemporal foci and the difficulty of visual detection in the heterogeneous neocortical landscape (Drzezga et al., 1999). Automated quantitative analysis tools such as statistical parametric mapping (SPM) and three-dimensional stereotactic surface projections (3D-SSP) have improved observer-independent detection, particularly in ETLE, by standardizing comparison against normative databases (Drzezga et al., 1999).

A key limitation of FDG-PET is its inability to precisely define surgical margins (Rathore et al., 2014; Vinton et al., 2007), as the hypometabolic region invariably exceeds the extent of the epileptogenic zone. Moreover, the presence of bilateral or extensive extratemporal hypometabolism has been associated with poorer postsurgical outcomes, potentially reflecting a more distributed epileptogenic network that is less amenable to focal resection. Timing of the scan relative to the last seizure is also crucial; postictal hypermetabolism can complicate interpretation if the interictal state has not been adequately established.

***Clinical bottom line:*** *interictal FDG-PET answers where interictal metabolic dysfunction lies and is most valuable in MRI-negative temporal lobe epilepsy, where regional hypometabolism can lateralize and guide SEEG even without a structural lesion. Because hypometabolism is typically more extensive than the epileptogenic zone, PET localizes a surrogate region rather than the zone itself and is best read alongside structural and electrophysiological data. Maturity: routine clinical use, particularly when co-registered to MRI*.

### 3.3 Ictal SPECT and SISCOM

Single-photon emission computed tomography (SPECT) is unique among presurgical imaging modalities in its capacity to capture a snapshot of regional cerebral blood flow (rCBF) at the time of seizure onset, thereby directly interrogating the ictal onset zone. Ictal SPECT relies on the rapid injection of a 99mTc-labeled perfusion radiotracer (HMPAO or ECD) within seconds of seizure onset, with subsequent imaging reflecting the distribution of cerebral perfusion at the moment of tracer uptake (O'Brien et al., 1998). Its diagnostic sensitivity for TLE is reported to be approximately 90%−97% for ictal studies, compared with only 44%−50% for interictal SPECT, underscoring the critical dependence on injection timing. In ETLE, ictal SPECT sensitivity is lower and more variable, typically in the range of 50%−70%, and is significantly influenced by the latency between seizure onset and tracer injection.

The introduction of subtraction ictal SPECT co-registered to MRI (SISCOM) by O'Brien et al. (1998) represented a major methodological advancement, transforming SPECT from a qualitative to a semi-quantitative technique. SISCOM involves voxel-by-voxel subtraction of the interictal from the ictal SPECT image, followed by statistical thresholding and co-registration onto the patient's structural MRI. This approach significantly enhances localization accuracy and reduces interobserver variability compared with conventional side-by-side visual analysis. Meta-analytic data have reported SISCOM positive rates of approximately 86%, with a concordance rate of 65% relative to the gold standard of intracranial EEG or surgical outcome (Chen and Guo, 2016). Notably, in MRI-negative patients, SISCOM retains a positive rate of approximately 84%, providing valuable localizing information in this particularly challenging subgroup. Concordant SISCOM localization has been associated with significantly better surgical outcomes, with seizure-free odds ratios of 3.28 compared with non-concordant cases (Chen and Guo, 2016). More recently, the PISCOM technique (PET-interictal subtracted ictal SPECT co-registered to MRI) (O'Brien et al., 1998), which substitutes interictal PET for interictal SPECT in the subtraction process, has been proposed as an alternative that may further enhance diagnostic performance by combining metabolic and perfusion information.

The practical limitations of ictal SPECT include the logistical demands of achieving rapid radiotracer injection during spontaneous seizures, the need for continuous video-EEG monitoring during the injection period (O'Brien et al., 1998), and the inherently lower spatial resolution compared with PET or MRI. Additionally, seizure propagation occurring before tracer fixation can result in hyperperfusion patterns that reflect propagation pathways rather than the true seizure onset zone, particularly in frontal lobe epilepsies characterized by rapid bilateral spread.

***Clinical bottom line:*** *Ictal SPECT, and particularly SISCOM, captures the early ictal perfusion change and is most useful in extratemporal and MRI-negative cases where interictal data are inconclusive. Its value is constrained by the logistics of ictal tracer injection and by sensitivity to injection latency, so a negative or delayed study does not exclude a focus. Maturity: specialized epilepsy-center use*.

### 3.4 Magnetoencephalography (MEG), magnetic source imaging (MSI), and electric source imaging (ESI)

Magnetoencephalography (MEG) records the tiny magnetic fields generated by neuronal postsynaptic currents, offering millisecond-level temporal resolution comparable to EEG but with a fundamentally different sensitivity profile. Because magnetic fields are minimally distorted by the skull and intervening tissue layers, MEG provides superior spatial accuracy for source localization, particularly for tangential cortical sources located within sulci—a geometry that generates relatively weak signals on scalp EEG (Bagić et al., 2011). When MEG dipole modeling is co-registered with structural MRI, the resulting magnetic source imaging (MSI) provides a powerful tool for mapping interictal epileptiform discharge (IED) generators.

The American Clinical MEG Society (ACMEGS) has recognized MSI as a non-redundant tool in presurgical evaluation, particularly valuable when standard modalities yield insufficient or discordant localizing information (Bagić et al., 2011). A systematic review and meta-analysis by Mouthaan et al. (2019). reported that MSI demonstrates sensitivity up to 82%−90% and specificity of approximately 53% for EZ localization, with a positive predictive value reaching 90% when concordant with intracranial EEG findings. The clinical utility of MEG spans a wide range across studies—sensitivity from 20 to 100% and specificity from less than 10 to 100%—reflecting substantial heterogeneity in study populations, analysis methods, and reference standards (Carrette and Stefan, 2019). In MRI-negative patients, MEG-detected IEDs have been identified in 32%−100% of cases across published series, providing critical localizing information that may not be obtainable from scalp EEG alone (Říha et al., 2022).

MEG is particularly advantageous for neocortical epilepsies, where its preferential sensitivity to tangential sources complements the radial source sensitivity of EEG, and for insular epilepsies where scalp EEG localization is notoriously challenging (Bagić et al., 2011; Carrette and Stefan, 2019). Because magnetic field strength decays rapidly with distance, signals arising from deep mesial temporal generators reach the sensor array attenuated. This should not be taken to mean that such sources are invisible to MEG: simultaneous MEG and intracranial recordings, and source analysis performed on the earliest resolvable phase of the discharge, have demonstrated that basal and mesial temporal discharges are detectable (Plummer et al., 2019). The practical limitation is therefore one of reduced localization accuracy rather than absent sensitivity, compounded by rapid propagation to lateral temporal neocortex, which the sensor array registers more readily than the mesial onset itself. Practical constraints include the high cost and limited availability of MEG systems, the requirement for magnetically shielded rooms, and the difficulty of capturing ictal events during the relatively short recording sessions. The recent development of optically pumped magnetometer (OPM)-based MEG systems promises to address some of these limitations by enabling wearable, movement-tolerant recordings outside shielded environments, although clinical validation in epilepsy cohorts is still in its early stages.

**Electric source imaging**. Electric source imaging (ESI) is the electroencephalographic counterpart of MSI, reconstructing the cortical generators of scalp-recorded epileptiform activity. Because MSI (from MEG) and ESI (from EEG) are simply source imaging applied to complementary aspects of the same neuronal currents, they are frequently performed together—either from simultaneous EEG/MEG recordings or from high-density EEG alone—and MSI and ESI are best understood as the magnetic and electric expressions of a single source-localization framework rather than as separate modalities. Prospective studies of ictal and interictal ESI have reported clinically useful localization, with accuracy improving substantially as electrode density increases (Mouthaan et al., 2019; Carrette and Stefan, 2019) and as an individualized head model derived from the patient's MRI is used. Notably, the way source imaging is combined with MRI differs from the coregistration of tomographic datasets, and more than one approach is in use. Most commonly, particularly for dipole-based solutions, a coordinate system is established on the individual T1-weighted volume using anatomical landmarks or a digitized scalp surface, onto which the reconstructed sources are then projected. Distributed source models, by contrast, yield voxel-wise maps of estimated activity that resemble the tomographic output of PET, SPECT, or fMRI and can therefore be overlaid on and analyzed alongside these modalities directly. ESI also lends itself naturally to fusion with MRI post-processing, and combinations of source imaging with morphometric analysis have been reported to improve the yield of presurgical evaluation, particularly in MRI-negative and extratemporal cases where MEG has value well beyond a simple tie-breaking role.

***Clinical bottom line:*** *MEG/MSI localizes interictal spike generators with high spatial and temporal precision and is especially valuable in neocortical, insular, and MRI-negative epilepsy, where it provides non-redundant localizing information that can directly shape SEEG coverage. Its sensitivity depends heavily on spike frequency and source orientation; deep mesial temporal sources are detectable but are localized with lower accuracy than neocortical ones. Maturity: specialized epilepsy-center use, supported by established clinical guidelines*.

### 3.5 Functional MRI (fMRI)

Functional MRI in the presurgical evaluation of epilepsy serves two complementary purposes: the mapping of eloquent cortex to guide safe surgical resection, and the investigation of epileptic network organization through analysis of blood-oxygen-level-dependent (BOLD) signal dynamics.

Task-based fMRI is now widely used for preoperative lateralization of language dominance as a non-invasive alternative to the Wada test (intracarotid amobarbital procedure). The strength of the supporting evidence differs appreciably between the language and memory applications, and warrants precise statement. The American Academy of Neurology practice guideline supports the use of fMRI in place of the intracarotid amobarbital procedure for language lateralization at Level C (Szaflarski et al., 2017). For memory, the guideline distinguishes two separate questions: presurgical fMRI of verbal memory or of language encoding should be considered for predicting verbal memory outcome (Level B), whereas the use of fMRI to lateralize memory function itself is supported only at Level C, and only in mesial temporal lobe epilepsy, being of unclear utility in other epilepsy types (Level U). Consistent with this weaker evidence base, fMRI-based memory lateralization is not part of routine practice at most centers, and the intracarotid amobarbital procedure retains a role where memory lateralization is decisive. Paradigms involving word generation, semantic decision, and verbal fluency have demonstrated high concordance rates (85%−95%) with Wada lateralization results in TLE, and fMRI-derived language maps are increasingly used to guide the extent of temporal lobe resection to minimize the risk of postoperative language deficits.

Simultaneous EEG-fMRI represents a more advanced application in which interictal epileptiform discharges recorded on EEG are used to model the hemodynamic response, revealing BOLD signal changes associated with spike generation. This technique can localize the irritative zone with high spatial resolution, particularly in focal cortical dysplasia, and has shown incremental value in patients with non-lesional MRI (van Houdt et al., 2013). However, EEG-fMRI is technically demanding, requiring artifact-corrected EEG recording within the MRI scanner, and its sensitivity is limited by the requirement for sufficient interictal discharges during the scanning session.

Resting-state fMRI (rs-fMRI) has emerged as an increasingly promising tool for characterizing epileptic networks without requiring task performance or EEG co-registration. By analyzing spontaneous low-frequency BOLD fluctuations, rs-fMRI can identify alterations in functional connectivity within and between canonical brain networks—such as the default mode network (DMN), salience network, and limbic network—that distinguish epileptic from non-epileptic tissue. A systematic review and meta-analysis by Chakraborty et al. reported an average concordance of 71.3% between independent component analysis (ICA)-based rs-fMRI localization and reference-standard localization of the seizure onset zone, with a pooled odds ratio of 2.63 (95% CI 0.66–10.56) that did not reach statistical significance (Chakraborty et al., 2020). While rs-fMRI offers the advantage of being widely accessible and easily acquired as part of a standard MRI session, its clinical translation remains limited by challenges in standardization, relatively low temporal resolution compared with electrophysiological methods, and the confounding influence of head motion artifacts.

***Clinical bottom line:*** *task-based f MRI is the principal non-invasive tool for language lateralization and has substantially reduced reliance on the Wada test for that purpose, although its accuracy falls in patients with atypical language dominance. Its role in memory is narrower: prediction of verbal memory outcome is the better-supported application, whereas memory lateralization rests on weaker (Level C) evidence confined to mesial temporal lobe epilepsy and is not routine practice. Resting-state f MRI for seizure-onset lateralization is promising but not yet reliable enough to define a resection target on its own. Maturity: task-based mapping in routine use; resting-state applications remain research tools*.

### 3.6 Diffusion tensor imaging (DTI) and structural connectivity

Diffusion tensor imaging (DTI) probes the microstructural integrity of white matter by measuring the anisotropic diffusion of water molecules along fiber tracts. In epilepsy, DTI has revealed widespread alterations in fractional anisotropy (FA), mean diffusivity (MD), and other tensor-derived metrics, not only within the suspected epileptogenic zone but also in remote white matter pathways, supporting the concept of epilepsy as a network disorder (Zhang et al., 2020).

The clinical utility of DTI in presurgical evaluation is primarily two-fold. First, tractography-based mapping of eloquent white matter tracts—particularly the arcuate fasciculus, corticospinal tract, and optic radiations (Yogarajah et al., 2009)—enables surgical planning that minimizes postoperative functional deficits. This is especially critical in cases where the epileptogenic zone abuts or overlaps with major fiber pathways. Second, quantitative DTI analyses can identify microstructural abnormalities in the perilesional white matter of FCD and other epileptogenic substrates, potentially refining the extent of the zone requiring resection.

Despite its growing role, DTI has notable limitations including sensitivity to crossing fibers (Yogarajah et al., 2009), partial volume effects, and limited specificity for distinguishing epileptogenic from non-epileptogenic white matter damage. Advanced diffusion models, including high angular resolution diffusion imaging (HARDI), diffusion kurtosis imaging (DKI), and neurite orientation dispersion and density imaging (NODDI), are being explored to overcome some of these limitations, but their clinical validation in epilepsy presurgical evaluation remains at an early stage.

***Clinical bottom line:*** *DTI and tractography answer a functional-risk question—which white-matter tracts must be preserved—and constitute an adjunctive planning tool rather than a localizing test, most useful for delineating optic radiations and language pathways before resection or ablation. They also supply the structural-connectivity substrate for the network analyses discussed in Section 6. Maturity: routine use for tract preservation; connectivity-based localization remains investigational*.

### 3.7 Arterial spin labeling (ASL)

Arterial spin labeling (ASL) MRI is a non-invasive perfusion technique that has gained increasing attention as a complement to nuclear medicine-based perfusion imaging. By magnetically labeling arterial blood water as an endogenous tracer, ASL provides quantitative maps of cerebral blood flow (CBF) without the need for radiotracer injection or radiation exposure. In the interictal state, hypoperfusion patterns on ASL often co-localize with FDG-PET hypometabolism, reflecting regions of reduced neuronal activity associated with the functional deficit zone (Boscolo Galazzo et al., 2016).

The integration of ASL into hybrid PET/MRI protocols has shown particular promise (Boscolo Galazzo et al., 2016), as the simultaneous acquisition of metabolic (PET) and perfusion (ASL) data within a single session provides complementary information. Studies have demonstrated substantial agreement between ASL hypoperfusion and FDG hypometabolism (kappa approximately 0.70), and the combination of both modalities can improve specificity and positive predictive value for TLE localization while preserving sensitivity. However, ASL has lower spatial resolution and signal-to-noise ratio compared with SPECT and PET, and its sensitivity for extratemporal foci remains inferior.

***Clinical bottom line:*** *ASL provides radiation-free perfusion mapping that co-localizes with FDG-PET hypometabolism and is an attractive adjunct in children and in centers without PET, although its lower signal-to-noise ratio and inferior performance in extratemporal foci limit standalone use. Maturity: emerging; a useful complement rather than a replacement for PET or SPECT*.

### 3.8 Invasive EEG: the intracranial reference standard

Although the focus of this review is non-invasive imaging, no account of presurgical evaluation is complete without situating intracranial EEG within the diagnostic spectrum, because it is the reference standard against which the non-invasive modalities discussed above are ultimately judged. When non-invasive data are concordant and point to a single resectable region, surgery may proceed without invasive monitoring; when they are discordant, insufficient, or implicate eloquent or deep structures, stereo-electroencephalography (SEEG) or subdural recording is used to test specific anatomo-electro-clinical hypotheses and to delineate the seizure onset zone directly (Li et al., 2023). Invasive EEG thus occupies the apex of the diagnostic hierarchy, providing the highest specificity for seizure onset but at the cost of sampling only the limited volume of tissue that the implanted electrodes reach.

This dependency reframes the purpose of multimodal fusion. Rather than competing with invasive EEG, non-invasive fusion serves to generate and rank the hypotheses that invasive EEG then tests, to guide safe and efficient electrode placement (Zhang et al., 2020; Rodionov et al., 2013), and—increasingly—to reduce the need for invasive monitoring in favorable cases. The relationship is therefore complementary and sequential: the quality of an SEEG implantation is only as good as the non-invasive hypotheses that inform it, which is precisely why accurate multimodal integration upstream has such a direct bearing on the diagnostic yield of invasive monitoring downstream.

***Clinical bottom line:*** *intracranial EEG (SEEG or subdural) remains the definitive test of seizure onset and the reference standard for the non-invasive modalities, but it is invasive, spatially limited by electrode coverage, and reserved for cases that non-invasive evaluation cannot resolve. The principal clinical contribution of multimodal fusion is to sharpen the hypotheses that determine whether, and where, invasive monitoring is performed. Maturity: routine and definitive, but invasive and hypothesis-dependent*.

**Figure 2:**
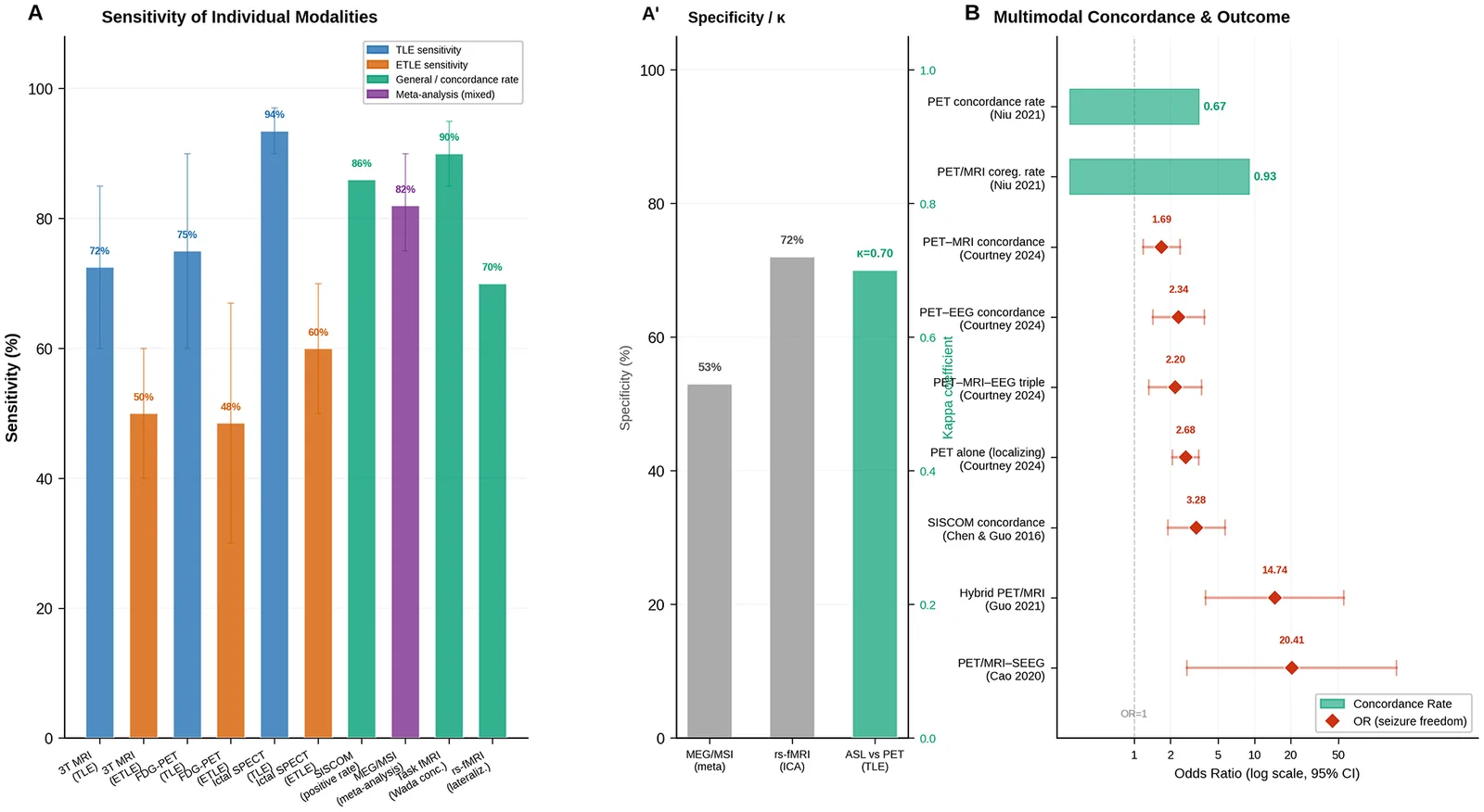
Diagnostic performance and prognostic value of neuroimaging modalities. **(A)** Reported sensitivity of individual modalities for temporal lobe epilepsy (TLE) and extratemporal lobe epilepsy (ETLE), alongside general concordance rates. **(A')** Specificity and kappa agreement metrics for selected modalities. **(B)** Multimodal concordance rates (teal bars) and associated odds ratios for postoperative seizure freedom (red diamonds with 95% confidence intervals), demonstrating that multimodality concordance significantly improves outcome prediction.

As this overview illustrates, each imaging modality provides a distinct yet incomplete window into the epileptic process. Structural MRI excels at lesion detection but is blind to functional abnormalities; FDG-PET captures metabolic dysfunction but lacks temporal precision; MEG offers exquisite temporal resolution for electrophysiological source localization but is limited by depth sensitivity; and ictal SPECT uniquely interrogates the seizure onset zone but is constrained by logistical complexity. The diagnostic gaps inherent in any single modality—particularly in MRI-negative cases where no single test reliably identifies the EZ—provide the fundamental rationale for multimodal neuroimaging fusion. The following section examines the principal technical approaches that have been developed to integrate information across these diverse imaging modalities into a coherent framework for presurgical decision-making. Figure 2 summarizes the comparative sensitivity of individual modalities and the prognostic value of multimodal concordance.

## 4 Technical approaches to multimodal neuroimaging fusion

The integration of information from multiple neuroimaging modalities into a unified spatial and interpretive framework is the central technical challenge of multimodal presurgical evaluation. Unlike sequential review of individual modality results—which relies on the clinician to mentally synthesize disparate data streams—multimodal fusion aims to systematically combine imaging outputs to yield localizing information that exceeds the diagnostic capacity of any single modality. This section reviews the principal technical strategies that have been developed for multimodal fusion in epilepsy presurgical evaluation, organized by level of integration complexity. Because several related terms are often used interchangeably, we distinguish them here. Co-registration refers to the spatial alignment of images into a common coordinate space; co-display denotes the visual overlay of co-registered images without further computation; concordance describes the qualitative agreement of independently interpreted modalities on a localization; hybrid acquisition refers to the simultaneous physical acquisition of two modalities on a single scanner (for example, PET/MRI); and machine-learning integration denotes the algorithmic combination of multimodal features into a single model output. In this review, “fusion” is used as the umbrella term for any of these, and the more specific term is used wherever the distinction is material.

### 4.1 Image coregistration: the foundation of multimodal integration

The most fundamental and widely practiced approach to multimodal fusion is spatial coregistration, in which images acquired from different modalities are aligned into a common coordinate space. In current clinical practice, the three-dimensional T1-weighted MRI volume (typically an MPRAGE sequence) serves as the anatomical reference or “base image,” onto which all other modalities—including FDG-PET, SPECT/SISCOM, MEG dipole source maps, fMRI activation maps, and DTI tractography—are coregistered (Jin et al., 2021).

Modern coregistration algorithms employ automated voxel-based methods, most commonly maximization of mutual information, which quantifies the statistical dependence between image intensities across modalities and iteratively adjusts spatial transformation parameters to maximize this dependence. For within-modality registration (e.g., aligning different MRI contrasts such as FLAIR to T1), linear rigid-body transformations are typically sufficient. Cross-modality registration between structurally dissimilar images (e.g., PET to MRI) may additionally require affine transformations to account for differences in voxel size and field of view. In cases involving significant brain deformation—such as postoperative imaging with a resection cavity—non-linear registration methods may be necessary.

**Registration models and pipelines**. The choice of registration model matters for how multimodal data are combined. For aligning different images from the same patient—for example, PET or SPECT to MRI—rigid-body registration with six degrees of freedom (three translations and three rotations) is standard, as it assumes the brain's shape is unchanged between acquisitions. Affine registration adds scaling and shearing (twelve degrees of freedom) and is useful when geometric distortion is present, as in echo-planar sequences. Deformable (non-linear) registration, which allows locally varying warps, is required when mapping an individual brain onto a population template for atlas-based parcellation or normative comparison, and for correcting the spatial distortions of diffusion and functional MRI. Registration is further divided into volume-based approaches, which operate on image intensities throughout the brain and are well suited to subcortical structures, and surface-based approaches, which align reconstructed cortical surfaces and underpin the surface-based feature extraction used by automated lesion-detection pipelines such as MELD. These registration steps typically sit within broader segmentation and parcellation pipelines—implemented in packages such as FreeSurfer, FSL, and SPM—that define the anatomical nodes on which quantitative and network analyses depend, so that errors introduced at the segmentation or registration stage propagate into every downstream fusion result. It should be noted that most of these tools were developed for research and that their translation into routine clinical use is constrained by regulatory requirements: relatively few dedicated multimodal fusion or lesion-detection platforms have obtained CE marking or FDA clearance, which remains a practical barrier to widespread clinical deployment.

The clinical impact of coregistration on diagnostic accuracy has been demonstrated in several settings. Salamon et al. (2008) reported that incorporating FDG-PET/MRI coregistration into the presurgical evaluation protocol at UCLA enhanced the non-invasive identification and successful surgical treatment of patients with cortical dysplasia, particularly benefiting the 33% of patients with non-concordant findings and those with normal MRI scans harboring mild type I cortical dysplasia. In a subsequent study of 103 patients with histologically confirmed FCD type 2, Desarnaud et al (2018) demonstrated that visual analysis of PET alone localized the lesion in only 44% of cases, whereas the addition of electroclinical data and PET/MRI coregistration increased the positive localization rate to 83%. The additional value of coregistration was particularly pronounced in patients with negative or equivocal MRI, localizing the FCD in 83% of this subgroup.

***Clinical bottom line:*** *co-registration is the technical foundation on which every fusion strategy depends; its accuracy sets the ceiling for all downstream interpretation, and registration error is an underappreciated source of apparent modality discordance. Maturity: routine, but quality control is essential and often insufficiently reported*.

### 4.2 Three-dimensional multimodality image integration (3DMMI)

Beyond two-dimensional overlay coregistration, three-dimensional multimodality image integration (3DMMI) represents a more advanced approach in which all available modality data are combined into an interactive 3D model of the individual patient's brain. This model simultaneously displays cortical surface anatomy, vascular structures, lesion locations, metabolic and perfusion abnormalities, electrophysiological source data, eloquent cortex maps, and planned or implanted electrode trajectories within a single visualization environment.

The Cleveland Clinic Epilepsy Center has developed and reported one of the most comprehensive clinical MMII workflows, implemented on the FDA-approved Curry platform (Compumedics Neuroscan). Jin et al. (2021) described the detailed methodology and reported on the utility of this approach from 2014 to 2018, during which 351 patients ultimately underwent surgery after MMII-guided evaluation. Among these patients, 56% had non-lesional MRI and 64% had extratemporal lobe epilepsy—a population composition that underscores the particular value of multimodal integration in challenging cases. Among 269 patients with at least 1 year of follow-up, 50% had remained completely seizure-free. The authors emphasized that the MMII workflow facilitates an iterative review process, in which concordant results from one modality can trigger focused re-review of another, sometimes transforming an initially negative MRI interpretation into a positive one when guided by electrophysiological localization data.

At University College London (UCL), Nowell et al. (2015) conducted a prospective study validating the use of 3DMMI in 44 patients undergoing intracranial EEG. This study employed a rigorous before-and-after design in which the multidisciplinary team made implantation decisions prior to disclosure of 3DMMI models, and any subsequent changes were systematically recorded. Disclosure of 3DMMI led to a change in overall implantation strategy in 34% of patients (15/44), including addition or subtraction of electrodes and decisions to proceed directly to resection. For detailed surgical planning, 3DMMI led to changes in 81% of patients (35/43), and 100% of patients undergoing SEEG had at least one electrode trajectory altered, with 75% of all 212 planned trajectories being modified. This landmark study provided robust evidence that multimodal integration substantially influences clinical decision-making in ways that cannot be achieved by sequential review of individual modality results.

The UCL group subsequently developed the EpiNav software platform, which combines image integration, 3D visualization, and computer-assisted planning (CAP) for SEEG electrode implantation. The complete 3DMMI pipeline—from image acquisition through coregistration, segmentation, 3D model construction, and trajectory planning—can be completed in approximately 1–2 h using this platform, compared with 2–4 h using previously available commercial software (Rodionov et al., 2013). More recently, the open-source SWANe (Standardized Workflow for Advanced Neuroimaging in Epilepsy) platform has been developed to provide a user-friendly Python-based tool for automated multimodal image processing and integration, with the goal of democratizing access to these techniques beyond specialized centers.

***Clinical bottom line:*** *three-dimensional multimodality integration is arguably the most immediately practical fusion application, because it directly shapes SEEG electrode trajectories, target selection, and avoidance of vasculature, and has been shown to change implantation strategy in a substantial proportion of cases. Its principal barrier is the need for a dedicated planning platform and trained personnel. Maturity: specialized epilepsy-center use*.

### 4.3 Concordance analysis and expert-based interpretation

In most epilepsy surgery programs, the clinical interpretation of multimodal imaging data is performed within a concordance framework by a multidisciplinary team at a patient management conference. Each modality result is reviewed independently, and the degree of spatial concordance among modalities is assessed qualitatively or semi-quantitatively. When multiple modalities converge on the same anatomical region, confidence in the localization hypothesis increases substantially; conversely, discordance between modalities signals uncertainty and often triggers additional investigations such as intracranial EEG.

This expert-based concordance approach, while intuitive and clinically pragmatic, has several inherent limitations. First, it depends heavily on the experience and composition of the multidisciplinary team, with potential for variability across centers. Second, qualitative concordance assessment does not account for the differing reliability and diagnostic accuracy of each modality in specific clinical scenarios—for example, FDG-PET has much higher sensitivity in temporal than extratemporal lobe epilepsy, and this differential performance should ideally be weighted in the concordance assessment. Third, the approach struggles to incorporate partial concordance or graded spatial overlap, instead typically dichotomizing results as concordant or discordant.

To address some of these limitations, Říha et al. (2022) conducted a systematic evaluation of multimodal combination methods in 25 patients with MRI-negative focal epilepsy using 38 imaging methods derived from 3T MRI, FDG-PET, HD-EEG, and SPECT. Their analysis identified a fundamental battery of 13 methods that optimized EZ localization accuracy while minimizing redundancy. The best-performing methods for localizing the presumed EZ in postoperative patients were SISCOM, FDG-PET, arterial spin labeling, regional homogeneity (ReHo) from resting-state fMRI, gray matter volume, cortical thickness, and high-density electrical source imaging. This work represents an important step toward evidence-based selection and combination of imaging modalities, although the relatively small sample size limits generalizability.

***Clinical bottom line:*** *expert-based concordance analysis remains the de facto standard by which multimodal data are integrated in clinical practice, and concordance across modalities is a consistent predictor of favorable outcome. Its limitation is that it is qualitative and observer-dependent—precisely the gap that quantitative and AI-based fusion aim to close. Maturity: routine, embedded in multidisciplinary conference review*.

### 4.4 Quantitative fusion strategies

Moving beyond qualitative concordance, several quantitative approaches have been proposed to systematically combine multimodal data.

**Voxel-based intersection and union methods**. The simplest quantitative approach involves computing the spatial overlap between thresholded abnormality maps from different modalities. The intersection (logical AND) of abnormal regions across modalities identifies voxels that are consistently abnormal, potentially delineating a region concordant with the presumed epileptogenic zone with high specificity but potentially low sensitivity. Conversely, the union (logical OR) captures the full extent of abnormality across modalities, maximizing sensitivity at the expense of specificity. These operations can be weighted by modality-specific confidence scores derived from the literature or from individual patient characteristics.

**Statistical parametric mapping and z-score integration**. Individual modalities can be converted into standardized z-score maps by comparison against normative databases, enabling direct quantitative comparison across modalities that measure fundamentally different biological signals. This approach has been applied to combine MRI morphometric maps (MAP, CPM), FDG-PET standardized uptake ratios, ASL cerebral blood flow maps, and SPECT perfusion data within a common statistical framework. The SISCOM technique itself is an early exemplar of this approach, converting ictal-interictal SPECT difference images into z-score maps referenced to the interictal standard deviation.

**Atlas-based and surface-based parcellation methods**. Rather than operating at the voxel level, modality results can be summarized within standardized anatomical parcellations (such as the Desikan-Killiany atlas or Talairach grid) and combined at the regional level. The Cleveland Clinic MMII workflow, for example, employs Talairach coordinates as a common spatial reference system for cross-modality comparison and SEEG planning (Jin et al., 2021). Surface-based methods using cortical parcellation from FreeSurfer or similar pipelines enable region-of-interest analyses that respect cortical topology.

***Clinical bottom line:*** *voxel-based and parametric fusion methods, such as statistical parametric mapping and z-score maps, offer reproducible, observer-independent integration and can improve detection of subtle abnormalities, but they integrate dipole-based ESI/MSI results poorly and depend on normative databases that may not match the individual patient. Maturity: specialized-center and research use*.

### 4.5 Simultaneous acquisition: hybrid PET/MRI

Hybrid PET/MRI systems represent the most technically integrated approach to multimodal fusion, enabling the truly simultaneous acquisition of metabolic PET and structural/functional MRI data within a single imaging session. Unlike software-based *post-hoc* coregistration—which combines images acquired at different times and in different scanners, introducing potential misregistration from differences in patient positioning—simultaneous PET/MRI provides inherently coregistered datasets with perfect spatial and temporal correspondence.

In the epilepsy context, hybrid PET/MRI offers several practical advantages beyond improved coregistration accuracy. The simultaneous acquisition of FDG-PET, high-resolution structural MRI, ASL perfusion, and fMRI within a single session reduces the total number of imaging appointments, minimizes patient burden (particularly important in pediatric populations), and eliminates intersession variability in brain state. Studies from multiple centers have confirmed that the diagnostic information obtained from the PET component of hybrid PET/MRI systems is comparable to that from dedicated PET/CT scanners, with the additional benefit of superior soft-tissue contrast from MRI and the avoidance of additional CT radiation.

The clinical value of hybrid PET/MRI in presurgical evaluation was examined by (Borbély et al. 2022) in a concordance analysis of 59 patients with discordant or MRI-negative refractory focal epilepsy. This study explored whether hybrid imaging could improve diagnostic classification compared with conventional sequential evaluation, and introduced a mathematical model for clinical concordance calculation. In non-lesional cases, hybrid FDG-PET/MRI revealed morphological lesions in 18 of 30 patients and glucose hypometabolism in 29 of 30 patients, demonstrating substantial incremental yield over conventional MRI alone (as reported in a separate study by the same group, see Section 2). While these results are promising, the high cost and limited availability of hybrid PET/MRI scanners currently restrict their use to specialized academic centers, and large-scale outcome studies comparing hybrid acquisition with conventional sequential approaches remain limited.

***Clinical bottom line:*** *simultaneous PET/MRI eliminates co-registration error, reduces patient burden and sedation exposure—particularly relevant in children—and enables single-session multimodal workups, with PET quantification comparable to PET/CT once attenuation correction is addressed. Its main barrier is capital cost and limited availability. Maturity: specialized epilepsy-center use, expanding*.

### 4.6 Emerging computational fusion approaches

Recent advances in computational methods are opening new frontiers for multimodal data fusion that go beyond spatial coregistration and concordance analysis.

**Joint independent component analysis (jICA) and linked ICA**. These methods decompose multimodal datasets into shared and modality-specific components, identifying common latent factors that drive covariation across modalities. In epilepsy, joint analysis of structural MRI and functional connectivity data has been used to identify coupled structural-functional abnormalities associated with the epileptogenic zone.

**Graph-based network fusion**. Building on the network perspective of epilepsy, graph-based methods can integrate structural connectivity (from DTI tractography) and functional connectivity (from rs-fMRI or MEG) into unified brain network models. Network metrics computed from these fused representations—such as node centrality, clustering, and modularity—may identify epileptogenic hub regions that are not apparent from any single modality alone.

**Deep learning-based fusion**. Convolutional neural networks and other deep learning architectures are increasingly being applied to combine multimodal imaging features for automated EZ localization. For example, dual-branch encoder networks have been proposed to jointly process T1-weighted MRI, FLAIR, FDG-PET, and morphometric post-processing maps for the detection of focal cortical dysplasia, achieving improved sensitivity compared with single-modality approaches. However, these methods currently require large training datasets that are difficult to assemble in the epilepsy surgery population, and their clinical validation remains at an early stage. The application of artificial intelligence to multimodal imaging is discussed in greater detail in Section 6.

***Clinical bottom line:*** *deep-learning-based fusion can in principle learn cross-modal representations that outperform hand-crafted integration, but current evidence is limited to proof-of-concept studies constrained by small, single-center datasets and minimal external validation. Maturity: research and experimental only*.

The evolution from simple visual overlay to sophisticated computational fusion reflects the increasing recognition that multimodal information must be integrated systematically, rather than merely displayed side-by-side. Importantly, the optimal fusion strategy may vary depending on the clinical scenario: hardware-level integration via hybrid PET/MRI is ideal when available but costly, 3DMMI provides the most comprehensive framework for surgical planning, and quantitative voxel-based approaches offer the promise of standardized, objective analysis. The following section examines how these fusion approaches have been applied in specific clinical contexts, focusing on the diagnostic performance of key modality combinations in the presurgical evaluation of drug-resistant epilepsy (see Table 2).

**Table 2:** Technical strategies for multimodal neuroimaging fusion in epilepsy presurgical evaluation.

Fusion strategy	Description	Key software/platforms	Advantages	Limitations
Spatial coregistration	Alignment of multimodal images to a common T1-weighted MRI reference using mutual information algorithms	SPM, FSL (FLIRT/FNIRT), FreeSurfer, 3D Slicer	Widely available; computationally straightforward; enables direct visual comparison	Dependent on registration accuracy; does not quantitatively combine information
3D multimodality integration (3DMMI)	Interactive 3D model combining cortical surface, vessels, modality maps, and electrode trajectories	Curry (Compumedics), EpiNav (UCL), SWANe, 3D Slicer with SEEG Assistant	Comprehensive visualization; supports SEEG planning; proven clinical impact (Nowell et al., 2015)	Time-intensive to build models; requires dedicated expertise; limited standardization
Concordance analysis	Qualitative or semi-quantitative assessment of spatial agreement across modality results by expert team	Clinical judgment at patient management conference	Integrates clinical context; flexible; standard of care	Subjective; not weighted by modality performance; binary concordant/discordant classification
Z-score normative mapping	Conversion of modality outputs to standardized z-scores via comparison with normative databases	SPM, MAP/CPM, SISCOM pipelines	Enables cross-modality quantitative comparison; observer-independent	Requires well-matched normative databases; sensitive to age/scanner variability
Atlas-based parcellation	Summarization of modality results within standardized anatomical regions (Talairach, Desikan-Killiany)	FreeSurfer, Talairach atlas tools	Reduces dimensionality; facilitates systematic regional comparison	Loss of sub-regional spatial information; dependent on parcellation scheme
Hybrid PET/MRI acquisition	Simultaneous acquisition of PET and MRI data in a single integrated scanner	Siemens Biograph mMR, GE SIGNA PET/MR	Inherently coregistered; reduced patient burden; simultaneous metabolic-structural data	High cost; limited availability; requires specialized technical expertise
Deep learning-based fusion	Neural networks combining multimodal features for automated EZ detection	Custom research architectures (e.g., DE-MAPGNet)	Can learn complex cross-modal relationships; potentially automated	Requires large datasets; limited clinical validation; “black box” interpretability

## 5 Clinical evidence for multimodal combinations in presurgical evaluation

The preceding sections have established the theoretical rationale and technical framework for multimodal neuroimaging fusion. This section examines the clinical evidence supporting specific modality combinations, focusing on their diagnostic yield and prognostic value for surgical outcome prediction. We organize this evidence by the principal modality pairings that have been most extensively studied.

### 5.1 MRI + FDG-PET: the core structural–metabolic combination

The combination of structural MRI and FDG-PET constitutes the most widely studied and clinically established multimodal pairing in epilepsy presurgical evaluation. These two modalities are complementary in a fundamental sense: MRI provides high-resolution structural characterization of potential lesions, while FDG-PET reveals metabolic dysfunction that frequently extends beyond the anatomical lesion boundary and can identify abnormalities in structurally normal-appearing cortex.

**Concordance and surgical outcome prediction**. The largest and most recent meta-analysis addressing this question was conducted by Courtney et al., published in *Neurology*. This systematic review encompassed 98 studies with a total of 4,104 patients and examined the association between localizing FDG-PET hypometabolism and surgical outcome. Localizing PET hypometabolism concordant with the resection zone was associated with favorable outcome (Engel class I or equivalent) with an odds ratio (OR) of 2.68 (95% CI 2.08–3.45). Critically, this association held for both MRI-positive patients (OR 2.64, 95% CI 1.54–4.52) and MRI-negative patients (OR 2.49, 95% CI 1.80–3.44), demonstrating that PET provides independent prognostic value regardless of MRI findings.

Concordance between PET and other modalities further enhanced outcome prediction. PET–EEG concordance yielded an OR of 2.34 (95% CI 1.43–3.83), PET–MRI concordance an OR of 1.69 (95% CI 1.19–2.40), and triple concordance among PET, MRI, and EEG an OR of 2.20 (95% CI 1.32–3.64). Conversely, diffuse PET hypometabolism was associated with significantly worse outcomes compared with focal hypometabolism (OR 0.34, 95% CI 0.22–0.54), highlighting the importance of hypometabolic pattern analysis in patient selection.

**PET/MRI coregistration improves localization**. The Niu et al. (2021) meta-analysis of 44 studies provided quantitative evidence for the superiority of PET/MRI coregistration over PET alone. The pooled concordance rate of FDG-PET with the reference standard was 0.67 (95% CI 0.60–0.73), while FDG-PET/MRI coregistration achieved a substantially higher concordance rate of 0.93 (95% CI 0.89–0.97). This dramatic improvement underscores the synergistic value of spatial integration beyond what either modality achieves independently.

**Cortical dysplasia detection**. The MRI–PET combination has demonstrated particular value in detecting focal cortical dysplasia (FCD), the most common histopathological substrate in extratemporal epilepsy surgery. In a study of 103 patients with histologically confirmed FCD type 2, Desarnaud et al. (2018) reported that visual PET analysis alone identified the lesion in only 44% of cases. When electroclinical data were integrated, this increased to 73%, and with the further addition of PET/MRI coregistration, the localization rate reached 83%. MRI was positive in 59% of this cohort, confirming PET's critical complementary role. The additional value of PET was most pronounced in MRI-negative or MRI-equivocal patients, with PET localizing the FCD in 83% of this subgroup.

### 5.2 MRI + SPECT/SISCOM: structural–hemodynamic integration

Ictal SPECT combined with structural MRI through the SISCOM technique represents one of the earliest and most clinically validated forms of multimodal fusion in epilepsy.

**Meta-analytic evidence**. The Chen and Guo (2016) meta-analysis of 11 SISCOM studies provided comprehensive performance data. The unweighted positive rate of SISCOM was 85.9%, with a concordance rate of 65.3% against the gold standard. In 142 MRI-negative patients specifically, the SISCOM positive rate was 83.8%, confirming its utility when structural imaging is unrevealing. Among 275 surgical patients, the seizure-free OR was 3.28 (95% CI 1.90–5.67) for concordant vs. non-concordant SISCOM results. For the extratemporal lobe epilepsy subgroup, the seizure-free OR remained significant at 2.44 (95% CI 1.34–4.43). The pooled positive predictive value of concordant SISCOM for favorable surgical outcome was 56%.

**Extratemporal epilepsy**. The prognostic value of SISCOM in extratemporal epilepsy was further supported by O'Brien et al. (1998), who demonstrated in 36 consecutive extratemporal epilepsy patients that 57.9% with SISCOM concordant with the surgical site achieved excellent outcome, compared with only 17.6% with non-concordant or non-localizing SISCOM (*p* < 0.05). Importantly, SISCOM was predictive of outcome independently of MRI or scalp ictal EEG findings in logistic regression analysis. Furthermore, the extent of resection of the SISCOM focus was significantly associated with outcome: 100% with complete resection, 60% with partial resection, and 20% with non-resection of the SISCOM focus achieved excellent outcomes.

**PISCOM as an emerging alternative**. As discussed in Section 2, PISCOM (PET-interictal subtraction coregistered to MRI), which substitutes interictal FDG-PET for interictal SPECT, has emerged as a promising alternative that eliminates the need for a separate interictal SPECT acquisition. Recent comparative data suggest that PISCOM demonstrates comparable sensitivity to SISCOM for EZ localization, though further validation in larger cohorts is needed before it can replace SISCOM in routine clinical practice.

### 5.3 PET/MRI + MEG: multimodal triangulation

The combination of metabolic (PET), structural (MRI), and electromagnetic (MEG) data represents a powerful triangulation strategy that samples complementary aspects of the epileptogenic zone.

Guo et al. (2022) conducted a retrospective study of 73 TLE patients who underwent both [18F]FDG PET/MRI and MEG, with surgical resection and outcome as the reference standard. MRI alone showed structural abnormality concordant with the resection site in 46.6% (34/73) of patients. FDG-PET with SPM analysis demonstrated concordant focal hypometabolism in 67.1% (49/73), which increased to 82.2% (60/73) with simultaneous MRI coregistration. MEG source localization was concordant with the resection in 71.2% (52/73). When all three modalities were combined, lobar localization was achieved in 94.5% (69/73) of patients—a significantly higher rate than PET/MRI or MEG alone (χ^2^ = 13.948, *p* < 0.001 vs. PET/MRI; χ^2^ = 5.393, *p* = 0.020 vs. MEG). The concordance of PET/MRI/MEG with surgical outcome was also significantly superior to that of either PET/MRI or MEG individually.

This study provides compelling evidence for the incremental value of adding MEG to the PET/MRI combination. MEG contributes non-redundant localizing information due to its unique sensitivity to tangentially oriented cortical sources and its millisecond temporal resolution, which captures epileptiform activity patterns not accessible to metabolic imaging. Notably, MEG's contribution was most pronounced in the subset of patients where PET/MRI localization was equivocal or discordant, suggesting that MEG serves a particularly important role as a “tiebreaker” modality.

### 5.4 Multimodal concordance and surgical outcome

Beyond specific modality pairings, a growing body of evidence demonstrates that the degree of overall multimodal concordance is itself a powerful predictor of surgical outcome. The principle is intuitive: when multiple independent modalities converge on the same localization, the probability that this represents the true epileptogenic zone is high, and consequently the likelihood of postoperative seizure freedom increases.

**The concordance–outcome relationship**. The Courtney et al. meta-analysis formalized this relationship by demonstrating graded odds ratios for different levels of concordance. Single-modality concordance (PET alone) yielded OR 2.68, dual concordance (PET + EEG) yielded OR 2.34, and triple concordance (PET + MRI + EEG) yielded OR 2.20. While the absolute OR values vary depending on the specific combination measured, the consistent finding across studies is that multimodal concordance significantly improves outcome prediction compared with any single modality.

**MRI-negative epilepsy**. Multimodal concordance assumes particular importance in MRI-negative epilepsy, where the absence of a visible structural lesion necessitates greater reliance on functional and metabolic modalities. The Cleveland Clinic MMII experience reported by Jin et al. (2021) is instructive: among 351 surgical patients evaluated with comprehensive multimodal integration, 56% had non-lesional MRI. The 50% seizure-free rate at 1 year in this cohort—which included a high proportion of challenging MRI-negative and extratemporal cases—compares favorably with historical series, suggesting that systematic multimodal integration can extend the benefits of epilepsy surgery to patients who might otherwise be excluded from surgical consideration.

In the Říha et al. (2022) study of 25 MRI-negative patients, the systematic evaluation of 38 imaging methods identified that SISCOM, FDG-PET, and ASL were the top-performing individual methods for EZ localization in MRI-negative epilepsy. Surface-based cortical thickness analysis and voxel-based gray matter volume measures also contributed meaningful localizing information. The combination of selected methods from this fundamental battery outperformed any single method, with optimized multimodal combinations achieving substantially higher localization accuracy than individual approaches.

### 5.5 Hybrid PET/MRI: simultaneous acquisition in clinical practice

Hybrid PET/MRI systems enable true simultaneous acquisition, eliminating temporal and spatial misregistration between modalities. Several clinical studies have examined the diagnostic yield of this approach.

Guo et al. (2021) assessed localization accuracy and postsurgical outcome prediction of simultaneous FDG-PET/MRI in 98 refractory epilepsy patients. Concordance between the resection area and the hypometabolic zone on hybrid PET/MRI predicted seizure freedom with an OR of 14.741 (95% CI 3.934–55.033, *p* < 0.001). The sensitivity of PET/MRI for identifying the seizure onset zone was 95.3%, although specificity was low at 8.8%, reflecting the known tendency for PET hypometabolism to extend beyond the seizure onset zone.

In the Zhang et al. (2020) Ruijin Hospital study of 42 refractory epilepsy patients undergoing SEEG implantation guided by hybrid PET/MRI, PET/MRI concordance with SEEG was found in 29 patients (69.05%), and concordant results between SEEG and PET/MRI were strongly associated with satisfactory surgical outcome (OR 20.41, 95% CI 2.75–151.4, p = 0.003). A subsequent study from the same group Li et al. (2023) extended this approach to 27 MRI-negative patients undergoing SEEG-guided radiofrequency thermocoagulation, further confirming the clinical utility of hybrid PET/MRI in guiding minimally invasive interventions. These results, while derived from a relatively small single-center cohort, suggest that hybrid PET/MRI may have particular utility in MRI-negative patients being evaluated for minimally invasive interventions.

### 5.6 Single-session multimodal protocols

An emerging trend is the development of single-session protocols that acquire multiple imaging modalities within a single patient visit using hybrid PET/MRI systems. (Grouiller et al. 2015) demonstrated the feasibility of a “quadrimodal” single-session protocol combining FDG-PET, structural MRI, EEG-fMRI, and EEG source imaging (ESI) in 12 patients using a hybrid PET/MR scanner with a high-density 256-electrode MR-compatible EEG system. The complete acquisition was performed in less than 2 h with good patient comfort and data quality, providing clinically contributory data from at least two modalities in 9 of 12 patients and from all four modalities in 5 patients.

Such single-session protocols offer substantial practical advantages: they reduce the total number of hospital visits, ensure identical medication and physiological states across all modalities, and minimize the cumulative burden of repeated sedation in pediatric patients. As hybrid PET/MRI technology becomes more widely available and standardized acquisition protocols are developed, single-session multimodal evaluation may emerge as a new paradigm for presurgical assessment.

### 5.7 Summary of clinical evidence

The clinical evidence reviewed in this section supports several key conclusions. First, multimodal combinations consistently outperform individual modalities for EZ localization and surgical outcome prediction, with the magnitude of improvement varying by clinical context. Second, PET/MRI coregistration represents a particularly high-yield combination, with meta-analytic concordance rates approaching 93% compared with 67% for PET alone. Third, the addition of MEG to the PET/MRI combination provides further incremental value, achieving lobar localization in approximately 95% of TLE patients. Fourth, multimodal concordance is itself an independent predictor of favorable surgical outcome, with concordant localizations associated with 2–3-fold higher odds of seizure freedom. Fifth, these benefits are particularly pronounced in the MRI-negative population, where multimodal integration is often the decisive factor in determining surgical candidacy. Table 3 summarizes the key clinical studies and their principal findings.

**Table 3:** Key clinical studies on multimodal imaging combinations in epilepsy surgery.

Study	Design	Population	Modality combination	Key finding
Courtney et al. (2024) (^*^Neurology^*^)	Systematic review and meta-analysis, 98 studies	*n* = 4,104 surgical patients	FDG-PET ± MRI ± EEG concordance	Localizing PET: OR 2.68 for favorable outcome; PET–MRI–EEG triple concordance: OR 2.20; diffuse hypometabolism: OR 0.34 (worse outcome)
Niu et al. (2021) (^*^Epilepsy Behav^*^)	Meta-analysis, 44 studies	Patients with epilepsy	FDG-PET vs. FDG-PET/MRI coregistration	PET concordance 0.67; PET/MRI coregistration concordance 0.93
Chen and Guo (2016) (^*^Seizure^*^)	Meta-analysis, 11 studies	*n* = 275 surgical patients	SISCOM (ictal SPECT + MRI)	Positive rate 85.9%; concordance 65.3%; seizure-free OR 3.28 concordant vs. non-concordant; MRI-negative positive rate 83.8%
Guo et al. (2022) (^*^Eur Radiol^*^)	Retrospective, single center	*n* = 73 TLE patients	PET/MRI + MEG	MRI concordant 46.6%; PET/MRI 82.2%; MEG 71.2%; PET/MRI/MEG 94.5% lobar localization
Desarnaud et al. (2018) (^*^EJNMMI^*^)	Retrospective, single center	*n* = 103 FCD type 2	FDG-PET + MRI coregistration	PET visual alone 44%; +electroclinical 73%; +PET/MRI coregistration 83%; MRI-negative/equivocal subgroup 83%
Jin et al. (2021) (^*^Front Neurol^*^)	Retrospective, single center	*n* = 351 surgical (56% non-lesional)	Comprehensive MMII (MRI + PET + SPECT + MEG)	50% seizure-free at 1 year; workflow supports iterative re-review
Guo et al. (2021) (^*^Eur Radiol^*^)	Retrospective, single center	*n* = 98 refractory epilepsy	Hybrid FDG-PET/MRI	Concordance with resection predicts seizure freedom: OR 14.741; sensitivity 95.3%, specificity 8.8%
Zhang et al. (2020)/Li et al. (2023)	Retrospective, single center	*n* = 42 (Cao)/*n* = 27 (Li), MRI-negative	Hybrid PET/MRI + SEEG	PET/MRI–SEEG concordance 69%: OR 20.41 for favorable outcome (Cao, *n* = 42); thermocoagulation feasibility confirmed (Li, *n* = 27)
Nowell et al. (2015) (^*^Epilepsia^*^)	Prospective, single center	*n* = 44 IC-EEG patients	3DMMI (MRI + PET + fMRI + DTI)	Strategy changed in 34%; planning changed in 81%; 75% of SEEG trajectories altered
Říha et al. (2022) (^*^Sci Rep^*^)	Retrospective, single center	*n* = 25 MRI-negative	38 methods from MRI, PET, SPECT, HD-EEG	Fundamental battery of 13 methods; SISCOM, PET, ASL top performers; multimodal combination superior to single methods

The accumulating evidence clearly demonstrates that multimodal neuroimaging integration, whether achieved through software-based coregistration, comprehensive 3D integration, or hardware-level hybrid acquisition, provides substantial and clinically meaningful improvements in EZ localization and outcome prediction across the spectrum of drug-resistant focal epilepsy. However, several important caveats apply: most evidence comes from retrospective single-center studies, sample sizes are often modest, methodological heterogeneity complicates cross-study comparison, and randomized controlled trials comparing multimodal vs. standard evaluation are lacking. The following sections examine how emerging analytical frameworks—including network analysis and artificial intelligence—are being applied to further enhance the utility of multimodal neuroimaging data.

### 5.8 A practical framework: recommended multimodal imaging strategy by clinical scenario

The preceding sections summarized the evidence for individual modality combinations. From a practical standpoint, however, the clinically relevant question is rarely which combination performs best in general, but rather which combination is most informative for a given patient. The optimal multimodal strategy depends on the clinical scenario—in particular on lesion status, temporal vs. extratemporal localization, patient age, and the specific decision at hand, whether lateralization, stereo-EEG (SEEG) planning, or functional-risk mapping. Table 4 distills the evidence reviewed above into a scenario-based framework, indicating for each common presurgical situation the recommended modality combination, its expected contribution, its principal limitation, and the current level of supporting evidence. We emphasize that this framework is intended to support, not to replace, the individualized judgment of a multidisciplinary epilepsy surgery conference, and that the levels of evidence assigned reflect the maturity of clinical validation rather than the technical sophistication of the modality.

**Table 4:** Recommended multimodal imaging strategy by clinical scenario.

Clinical scenario	Recommended modality combination	Expected contribution	Principal limitation	Level of evidence
MRI-positive temporal lobe epilepsy	MRI + FDG-PET	Confirmation and lateralization of a concordant focus	Fusion adds limited incremental value when MRI is already concordant	High
MRI-negative temporal lobe epilepsy	PET/MRI + MEG/ESI + MRI post-processing (MAP)	Reveals occult metabolic or electromagnetic focus beyond structural imaging	False positives; usually requires SEEG confirmation	Moderate
MRI-negative extratemporal epilepsy	PET/MRI + ictal SPECT/SISCOM + MEG + 3DMMI	Narrows candidate region and constrains SEEG hypotheses	Higher localization uncertainty; frequently non-lesional	Moderate
Suspected focal cortical dysplasia	3T HARNESS + MRI post-processing (MAP) ± 7T + AI-based detection	Detects subtle or visually occult dysplasia	High false-positive rate; detections require validation	Moderate
Discordant non-invasive data	Full multimodal fusion + 3DMMI feeding SEEG planning	Reconciles competing localizing hypotheses	Strongly dependent on expert interpretation	Low–Moderate
Suspected insular epilepsy	MEG/ESI + FDG-PET + SEEG	Localizes deep or hidden seizure onset	Scalp EEG unreliable; deep sources remain challenging	Low–Moderate
Pediatric drug-resistant epilepsy	MRI + FDG-PET ± ASL	Detects FCD while limiting radiation exposure	Limited pediatric-specific validation data	Low–Moderate
SEEG implantation planning	3DMMI integrating all available non-invasive data, including MSI/ESI where available	Guides electrode trajectories and targets; avoids vasculature	Requires a dedicated fusion/planning platform	Moderate
Proximity to eloquent cortex	Task-based fMRI + DTI tractography ± task-based MEG	Maps eloquent cortex and white-matter tracts to preserve	Adjunctive functional-risk tool, not EZ-localizing	Moderate

## 6 Network-based approaches to multimodal neuroimaging in epilepsy

### 6.1 Conceptual shift: from epileptic focus to epileptic network.

**Figure 3:**
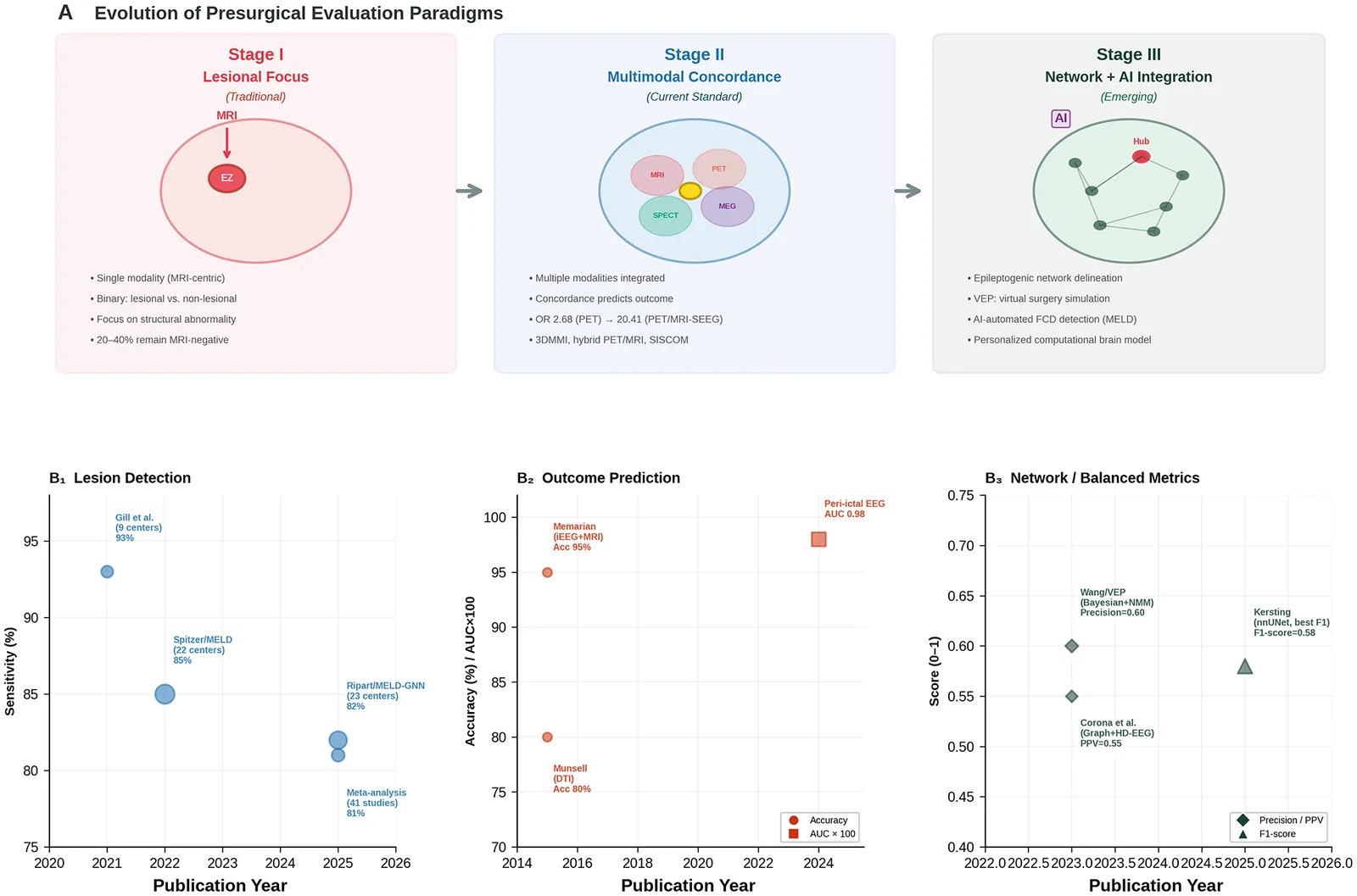
Evolution and performance of advanced analytical paradigms. **(A)** Conceptual shift from the traditional single-modality lesional focus model (Stage I), through current multimodal concordance frameworks (Stage II), to emerging network and artificial intelligence integration (Stage III). **(B1–B3)** Quantitative performance benchmarks of key AI and network-based studies over time, categorized by application: **(B1)** automated lesion detection sensitivity, **(B2)** surgical outcome prediction accuracy/AUC, and **(B3)** network-based or balanced detection metrics (Precision/PPV and F1-score).

Figure 3A illustrates the evolution of presurgical evaluation paradigms, from a zone-based conception in which a circumscribed epileptogenic region is inferred from converging surrogate markers, through multimodal concordance, to distributed-network and AI-integrated approaches. The contrast drawn in Panel A is between these conceptual frameworks rather than between lesional and non-lesional epilepsy: the classical model applies equally to non-lesional cases, and a lesion seen on MRI is never taken as epileptogenic without electrophysiological confirmation. Figure 3B presents the performance of key AI/ML studies across different analytical domains.

The traditional model of focal epilepsy posits a discrete cortical “focus” from which seizures originate and subsequently propagate. This conceptual framework directly informed the presurgical evaluation paradigm described in previous sections, where the primary goal is to localize the epileptogenic zone (EZ) as precisely as possible for targeted resection. However, accumulating evidence from both clinical and basic neuroscience has prompted a fundamental reconceptualization: epilepsy is increasingly understood as a network disorder in which seizures emerge from the dynamic interactions among distributed but interconnected brain regions rather than from a single discrete generator.

This paradigm shift, formally recognized by the International League Against Epilepsy (ILAE) in 2010 when the term “network” was incorporated into the revised classification of seizures and epilepsies, has profound implications for multimodal neuroimaging. Rather than simply identifying the “where” of seizure onset, network-based approaches seek to characterize the “how” of seizure generation and propagation—mapping the structural and functional connections that facilitate ictal dynamics and identifying critical network nodes whose disruption could terminate seizure activity. Multimodal neuroimaging is uniquely positioned to support this network perspective, as different imaging modalities capture complementary aspects of brain connectivity: structural MRI and DTI characterize the anatomical scaffold, rs-fMRI reveals functional coupling patterns, EEG/MEG provide temporal dynamics of neural synchronization, and PET maps metabolic network dysfunction.

A clarification of scope is warranted here, because the relationship between network analysis and the multimodal fusion that is the subject of this review is easily conflated. Network analysis is not intrinsically multimodal: a connectome can be constructed from a single modality, such as resting-state fMRI or MEG alone. It becomes a multimodal undertaking only when structural connectivity from diffusion MRI is combined with functional or metabolic information, or when network metrics are integrated with the structural, metabolic, and electrophysiological markers discussed in earlier sections. We therefore regard network characterization not as a competing paradigm that supersedes zone-based localization, but as an additional layer of information to be fused with the others—one that describes the organization and propagation properties of the epileptic system rather than a single point of origin.

This framing also invites a necessary note of caution. The clinical success of focal resective surgery, which remains predicated on removing a single circumscribed region, is itself strong evidence that a focal conception of the epileptogenic zone retains substantial validity. If a network hub can be surgically removed to achieve seizure freedom, it is reasonable to ask how such a hub differs operationally from the conventional epileptogenic zone, and whether the network abnormalities detected by imaging are genuinely epileptogenic in the sense of Rosenow and Lüders—that is, necessary and sufficient for seizure generation—or are instead downstream consequences of recurrent seizures. Distinguishing epileptogenic network nodes from nodes that are merely altered by the disease remains an unresolved and clinically consequential question that current cross-sectional imaging is not well positioned to answer.

### 6.2 Graph theory framework for epileptic networks

Graph theory provides the mathematical framework for analyzing brain networks as collections of nodes (brain regions) connected by edges (structural or functional connections). In epilepsy research, graph-theoretic analysis has been applied to networks derived from multiple imaging modalities, yielding several converging insights.

**Key network metrics**. The most commonly employed graph metrics in epilepsy network analysis include degree centrality (the number of connections a node has), betweenness centrality (the proportion of shortest paths passing through a node), nodal strength (the sum of weighted connections), clustering coefficient (the tendency of neighbors to be interconnected), and modularity (the degree to which the network can be partitioned into distinct communities). Hub nodes—regions with disproportionately high connectivity or centrality—are of particular interest because they may serve as critical facilitators of seizure propagation. In the context of epileptic networks, hub nodes are typically identified as those ranked in the top 5%−10% by degree centrality or betweenness centrality.

Functional connectivity changes in epilepsy. Studies using resting-state f MRI and MEG have consistently demonstrated altered network topology in patients with focal epilepsy compared with healthy controls. In temporal lobe epilepsy (TLE), graph theory analysis of rs-f MRI connectivity has revealed higher global efficiency, lower clustering coefficient ratios, and shorter path lengths compared with controls—indicating a more synchronized but less segregated network architecture. A trend toward functional reorganization with contralateral hemispheric hub shifts has been observed, suggesting that the epileptic process induces large-scale network reconfiguration that extends far beyond the seizure onset zone.

### 6.3 Hub resection and surgical outcome

A critical translational question is whether the identification and surgical resection of network hubs improves postoperative seizure outcome. Several studies have directly addressed this question using different imaging modalities.

**MEG-derived hubs**. Ramaraju et al. (2020) studied 31 patients with drug-refractory focal epilepsy who underwent resting-state MEG and structural MRI as part of presurgical evaluation. Using graph analysis of MEG functional connectivity, the authors identified network hubs and assessed whether their inclusion in the surgical resection was associated with seizure freedom. The results demonstrated that removal of interictal MEG-derived network hubs was significantly associated with postoperative seizure freedom, supporting the concept that hub disruption may be mechanistically important for achieving seizure control. This finding was consistent with earlier MEG work by Nissen et al. (2017), who found that network hubs were located within the resection area in 9 of 14 seizure-free patients but in none of 8 patients who were not seizure-free.

**Non-invasive network mapping**. Corona et al. (2023), published in *Brain*, made a significant methodological advance by demonstrating that epileptogenic networks could be mapped non-invasively using high-density EEG and MEG source imaging with virtual electrode implantation. In 37 children and young adults with DRE, the authors computed functional connectivity using amplitude envelope correlation and phase locking value, generated minimum spanning tree networks, and calculated graph centrality measures for each virtual sensor. For good-outcome patients (*n* = 22, ILAE class I), functional connectivity was significantly higher inside than outside the resection area across multiple frequency bands and both modalities, a pattern not observed in poor-outcome patients (*n* = 15, ILAE class II–VI). Graph centrality measures were predictive of both the EZ and surgical outcome, with positive and negative predictive values ≥55% validated by leave-one-out cross-validation, outperforming conventional dipole-based source localization on spike data. Critically, these network measures maintained predictive value even on spike-free data segments, suggesting that the epileptogenic network's topological signature persists in the interictal background.

Intracranial EEG-derived networks. Rijal et al. (2023), published in Scientific Reports, extended this work to intracranial EEG recordings in 31 children with DRE (22 good outcome, 9 poor outcome). They demonstrated a hierarchical epileptogenic organization of nodal strength across brain states: functional connectivity was lower during interictal and pre-ictal states and progressively higher during ictal and post-ictal states. Resection of nodes with high functional connectivity was predictive of surgical outcome, with positive and negative predictive values ranging from 47 to 100% depending on the frequency band and brain state analyzed.

**Spike propagation mapping**. Matarrese et al. (2023), also published in *Brain*, revealed the relationship between interictal spike propagation and effective connectivity in 43 children with DRE who underwent intracranial monitoring. Using spatiotemporal mapping of spike propagation in source space, the authors identified spike onset zones, early-spread zones, and late-spread zones. In patients with good surgical outcome (*n* = 25, Engel I), the spike onset zone showed higher overlap with the resection area (96%) than the early-spread (86%, *p* = 0.01) or late-spread zones (59%, *p* = 0.002). Directional information flow analysis revealed that in 66% of good-outcome patients, effective connectivity flowed from onset to early-spread regions, whereas in 50% of poor-outcome patients, the flow was reversed (from early-spread to onset). Resection of the spike onset zone, but not the area of spike spread or the clinically defined seizure onset zone, predicted outcome with a positive predictive value of 79% and negative predictive value of 56% (*p* = 0.04).

### 6.4 Personalized computational brain models: the virtual epileptic patient

Perhaps the most ambitious application of network neuroscience to epilepsy surgery is the development of personalized whole-brain computational models that simulate individual patients' seizure dynamics and predict surgical outcomes through “virtual surgery.”

**The VEP workflow**. Wang, Jirsa, and colleagues at Aix-Marseille Université developed the Virtual Epileptic Patient (VEP) framework, published as a cover article in *Science Translational Medicine* in 2023. The VEP workflow constructs a patient-specific whole-brain network model from anatomical T1-weighted MRI (for brain parcellation) and diffusion-weighted MRI (for structural connectivity estimation). Each node in this network is equipped with a neural mass model (the Epileptor) capable of transitioning between normal and seizure states. Bayesian inference methods then fit the model parameters to the patient's actual SEEG seizure recordings, thereby estimating the epileptogenicity of each brain region and delineating the epileptogenic zone network (EZN).

**Retrospective validation**. The VEP workflow was evaluated retrospectively in 53 patients with drug-resistant focal epilepsy who underwent SEEG. The VEP reproduced clinically defined EZNs with a precision of 0.6, and the physical distance between VEP-identified and clinically defined epileptogenic regions was small. In the 25 patients who proceeded to surgery, VEP demonstrated significantly lower false discovery rates in seizure-free patients (mean 0.028) compared with non-seizure-free patients (mean 0.407), indicating that the model accurately identified the regions whose resection was necessary for seizure freedom and, conversely, that non-seizure-free patients had VEP-identified epileptogenic regions that remained unresected.

Prospective clinical trial. Based on these retrospective results, the VEP is currently being evaluated in the EPINOV multicenter clinical trial (NCT03643016), which aims to prospectively enroll 356 patients with drug-resistant focal epilepsy. This trial represents one of the first large-scale prospective evaluations of computational brain modeling in epilepsy surgery and, if positive, could establish personalized brain modeling as a standard component of presurgical evaluation. Until EPINOV and comparable trials report, however, the VEP and related “digital brain twin” frameworks should be regarded as investigational: their retrospective validation is encouraging but does not yet establish that model-guided surgery improves seizure outcome relative to standard evaluation, and their readiness for routine clinical use has not been demonstrated.

### 6.5 Multimodal integration in network analysis

The network analysis framework naturally lends itself to multimodal data integration, as different imaging modalities provide complementary views of the brain's network organization.

**Structure–function relationships**. A fundamental question in network neuroscience is the relationship between structural connectivity (the physical white matter pathways connecting brain regions) and functional connectivity (the statistical dependencies in neural activity between regions). In epilepsy, this relationship is particularly relevant because structural connectivity provides the anatomical scaffold along which seizures propagate, while functional connectivity captures the dynamic patterns of aberrant synchronization. DTI-derived structural networks and rs-fMRI-derived functional networks can be jointly analyzed to identify regions where structure–function coupling is disrupted, potentially indicating epileptogenic tissue. The VEP approach described above explicitly leverages this structure–function relationship by using structural connectivity to constrain a dynamical model that is then fitted to functional data (SEEG).

**Connectome-based analysis**. Recent studies have combined structural and functional connectivity analyses to improve outcome prediction. For instance, analysis of gray matter abnormalities from T1-weighted MRI and white matter abnormalities from diffusion-weighted MRI demonstrated that combining both types of abnormality improved surgical outcome discrimination (gray matter AUC = 0.68, white matter AUC = 0.65, combined union AUC = 0.72), and 100% of patients who had all concordant abnormal regions resected achieved favorable outcomes (ILAE class 1 or 2). This finding reinforces the principle that the epileptogenic network has both structural and functional signatures, and that comprehensive resection of multimodally concordant abnormal tissue optimizes seizure outcome.

### 6.6 Challenges and limitations of network approaches

Despite their considerable promise, network-based approaches face several significant challenges that currently limit their clinical translation.

**Methodological variability**. There is no standardized methodology for constructing epileptic brain networks. Choices regarding brain parcellation scheme, connectivity metric (amplitude correlation, phase locking, coherence, Granger causality, transfer entropy), frequency band, threshold selection, and graph metric all influence the resulting network characterization. This methodological heterogeneity makes it difficult to compare results across studies and to establish generalizable network biomarkers.

**Spatial coverage limitations**. Intracranial EEG-based network analysis, while offering high temporal resolution and signal quality, is inherently limited by electrode coverage. Any network analysis can only characterize the portion of the epileptogenic network that lies within the spatial sampling of implanted electrodes. This limitation can be partially addressed by non-invasive approaches (high-density EEG and MEG source imaging), but these sacrifice spatial resolution and signal-to-noise ratio.

**Computational complexity**. Personalized brain modeling approaches such as VEP require substantial computational resources and expertise. The Bayesian inference procedures for fitting model parameters are computationally intensive, and the workflow requires integration of multiple imaging modalities (structural MRI, diffusion MRI, SEEG), specialized software, and mathematical modeling expertise not typically available in clinical epilepsy centers.

**Validation requirements**. Most network studies are retrospective, with modest sample sizes from single centers. The relationship between network metrics and surgical outcome, while promising, has shown inconsistency across studies—for example, Nissen et al. (2018) found that MEG-derived network measures were not effective predictors in a larger 94-patient cohort, despite earlier positive results in smaller samples. Prospective multicenter validation, such as the ongoing EPINOV trial for VEP, is essential before network-based approaches can be incorporated into clinical decision-making.

### 6.7 Future directions: toward clinical network-guided surgery

The emerging concept of “epileptic network neurosurgery” (ENN) envisions a future paradigm where surgical planning is guided not only by traditional zone-based localization but also by comprehensive network characterization. Under this framework, presurgical evaluation would involve constructing patient-specific epileptic connectomes from multimodal neuroimaging data, identifying hub nodes and critical propagation pathways through graph-theoretic analysis, simulating the effects of different surgical strategies (resection, disconnection, or neuromodulation) through personalized computational models, and selecting the intervention strategy that optimally disrupts the epileptogenic network while preserving functional connectivity. It must be emphasized, however, that this remains a vision rather than established practice: no prospective study has yet demonstrated that network-guided resection improves seizure freedom beyond what is achieved by conventional zone-based localization, and resective surgery in current practice continues to target focal removal of a putative epileptogenic zone.

This vision requires advances on multiple fronts: standardization of network construction methodologies, development of clinically accessible computational tools, prospective validation in large multicenter cohorts, and integration of network analysis into the multidisciplinary team workflow alongside traditional clinical expertise. With the ongoing maturation of both computational methods and hybrid neuroimaging technology, network-guided epilepsy surgery may transition from a research paradigm to a clinical reality within the coming decade. We note, however, that “standardization” in this context may not imply a single universal pipeline. Because network analysis yields a multitude of metrics whose usefulness depends on the specific question—lateralization, hub identification, or propagation modeling—rigorous standardization will likely require defining the clinical question first and validating a pipeline against that question, rather than adopting one analysis for all purposes.

***Clinical bottom line:*** *the network paradigm has reshaped how epilepsy is conceptualized and provides a coherent framework for integrating structural, functional, and metabolic connectivity. Its clinical translation, however, remains largely aspirational: hub-resection and computational-model studies are mostly retrospective, single-center, and methodologically heterogeneous, at least one large study has found network metrics non-predictive, and no prospective evidence yet shows that network-guided surgery outperforms conventional localization. At present, network measures are best used as a complementary, hypothesis-generating layer within multimodal evaluation rather than as a standalone determinant of surgical targets. Maturity: predominantly research, with early prospective evaluation underway (EPINOV)*.

## 7 Artificial intelligence and machine learning integration in multimodal epilepsy neuroimaging

### 7.1 Overview: the AI imperative in epilepsy imaging

The complexity and volume of multimodal neuroimaging data generated during presurgical evaluation of drug-resistant epilepsy (DRE) pose significant challenges for clinical interpretation. Each imaging modality produces distinct data types—volumetric structural images, diffusion tensors, metabolic maps, hemodynamic time series, electromagnetic source distributions—that must be synthesized into a coherent clinical picture. Artificial intelligence (AI) and machine learning (ML) methods offer transformative potential for this task, enabling automated lesion detection, quantitative feature extraction, multimodal data fusion, and outcome prediction at scales and sensitivities beyond human visual analysis.

The application of AI in epilepsy neuroimaging has progressed rapidly over the past decade, evolving from traditional machine learning approaches using hand-crafted features to deep learning architectures that learn directly from raw imaging data. This section reviews the major applications of AI/ML across four domains: automated lesion detection, surgical outcome prediction, multimodal data integration, and emerging computational frameworks.

### 7.2 Automated lesion detection: focal cortical dysplasia as a paradigm

Focal cortical dysplasia (FCD) represents the ideal testing ground for AI-based lesion detection in epilepsy. FCDs are the third most common cause of drug-resistant focal epilepsy, surgical resection yields seizure freedom in up to 70% of eligible candidates, and accurate MRI detection is the strongest clinical predictor of favorable surgical outcome. Yet FCDs—particularly type I and subtle type II lesions—frequently escape visual detection on conventional MRI, with reported MRI-negative rates ranging from 20% to over 50% in surgical series. This detection gap represents a critical unmet clinical need where AI can make a measurable impact.

**Deep learning approaches**. Gill et al. (2021), published in *Neurology*, performed the first multicenter validation of a deep learning algorithm for FCD detection. Using clinically acquired 3D T1-weighted and 3D FLAIR MRI from 148 patients with histologically verified FCD at 9 centers, they trained a deep convolutional neural network (CNN) classifier incorporating Bayesian uncertainty estimation for risk stratification. The overall sensitivity was 93% (137/148 FCD detected) using leave-one-site-out cross-validation, with an average of 6 false positives per patient. Crucially, sensitivity in patients initially deemed MRI-negative was 85%, and in 73% of patients the FCD was among the clusters with the highest confidence. In an independent test cohort of 23 patients, sensitivity was 83% with an average of 5 false positives per patient, and specificity was 89% in healthy and disease controls. This study provided Class III evidence that deep learning on multimodal MRI can accurately identify FCD in patients previously diagnosed as MRI-negative.

**The MELD Project**. The Multi-center Epilepsy Lesion Detection (MELD) Project represents the largest collaborative effort in AI-based FCD detection. Spitzer et al. (2022), published in *Brain*, collated MRI data from 1,015 participants (618 FCD patients, 397 controls) across 22 epilepsy centers on 5 continents. Using 33 surface-based cortical features, they trained an interpretable neural network classifier. On a restricted “gold-standard” subcohort of seizure-free patients with FCD type IIB who had both T1 and FLAIR data, the MELD algorithm achieved a sensitivity of 85%. Across the entire withheld test cohort, sensitivity was 67% (59% without border zone adjustment), and specificity was 54%. A distinctive strength of this study was its interpretability: the pipeline generated individual patient reports identifying the location of predicted lesions, their abnormal imaging features, and the saliency of each feature to the classifier decision.

The MELD project subsequently advanced to graph neural network (GNN) architectures. Ripart et al. (2025), published in *JAMA Neurology*, trained the MELD Graph on data from 23 centers (703 FCD patients, 482 controls). In the test dataset, the MELD Graph achieved a sensitivity of 81.6% in histopathologically confirmed seizure-free patients and 63.7% in MRI-negative FCD patients, with a positive predictive value (PPV) of 67% (70% sensitivity, 60% specificity). In an independent test cohort of 116 patients, the PPV increased to 76% (72% sensitivity, 56% specificity), representing a significant improvement over the baseline algorithm (PPV 46%, 77% sensitivity, 47% specificity).

Systematic evidence synthesis. Dashtkoohi et al. (2025) conducted a systematic review and meta-analysis (2025) evaluating 41 studies (24 ANN-based, 17 traditional ML) on AI-based FCD detection from MRI. Meta-analysis of internal validation datasets showed a pooled sensitivity of 0.81 and specificity of 0.92 for AI models in detecting FCD lesions, establishing a benchmark for future algorithm development. These pooled estimates must, however, be interpreted with caution. They are derived predominantly from internal validation datasets, in which training and test data share the same scanner, protocol, and case mix, and they therefore substantially exceed the performance observed when the same class of algorithms is evaluated on independent, prospective, or multicenter data. This largely explains why the meta-analytic sensitivity of 0.81 and specificity of 0.92 is appreciably higher than the real-world figures reported by the MELD Graph and by the head-to-head comparison of Kersting et al. (2025); the pooled numbers are best read as an upper bound achievable under favorable conditions rather than as accuracy deployable across epilepsy centers. The comparison is further complicated by heterogeneity in the reference standard used to define a true lesion—histopathologically confirmed FCD, the resection zone, or radiological consensus—which is not uniform across the pooled studies.

**Comparative multicenter evaluation**. Kersting et al. (2025), published in *Epilepsia*, performed the first head-to-head comparison of six AI models (three established: MAP18, MELD, deepFCD; three newly trained: 2D-nnUNet, FastSurferCNN, 3D-nnUNet) on independent multicenter test data from 329 subjects across 4 epilepsy centers. The 3D-nnUNet achieved the highest balanced performance (F1 score 0.58, detection rate 55%, specificity 86%), while deepFCD had the highest detection rate (82%) but zero specificity. MELD showed the most consistent cross-center performance with detection rates between 46 and 54%. This study highlighted that FCD detection benefits from 3D input data and that no single algorithm yet achieves universally optimal performance.

### 7.3 Surgical outcome prediction

Beyond lesion detection, ML methods have been applied to predict surgical outcomes using multimodal presurgical data, potentially enabling more informed surgical candidacy decisions.

**Multimodal feature integration**. Memarian et al. (2015) demonstrated that combining electrophysiological features from intracranial EEG, quantitative MRI measures of gray matter thickness, and clinical/demographic variables using a least-square support vector machine (LS-SVM) classifier achieved 95% surgical outcome prediction accuracy in mesial temporal lobe epilepsy (MTLE)—significantly outperforming any single modality alone. Maximum relevance minimum redundancy (mRMR) feature selection identified the most informative predictors: family history of epilepsy, ictal EEG onset pattern, MRI-based gray matter thickness, proportion of seizures originating in the ipsilateral amygdala, age, and epilepsy duration.

**Connectome-based prediction**. Munsell et al. (2015) evaluated ML algorithms for surgical outcome prediction using structural brain connectome data derived from presurgical DTI in patients with TLE. Using a two-stage connectome-based prediction framework with elastic net feature selection and SVM classification, the algorithm separated TLE patients from controls with 80% accuracy and predicted surgical outcome with 70% accuracy. Gleichgerrcht et al. (2020), published in *Annals of Neurology*, further demonstrated that TLE surgical outcomes can be inferred from structural connectome hubs identified through machine learning.

**Peri-ictal EEG analysis**. Sheikh et al. (2024), in a 2024 study in *Scientific Reports* using a dataset of 294 patients who underwent temporal lobe resection showed that ML classifiers could predict postoperative seizure outcome using just 5 min of peri-ictal scalp EEG data with an AUC of 0.98 and out-of-group testing accuracy exceeding 90%, suggesting that even routinely acquired non-invasive data may contain rich prognostic information when analyzed with appropriate computational tools.

**Dimensional disease modeling**. Bernasconi et al. (2025) highlighted unsupervised ML approaches, such as latent Dirichlet allocation applied to multimodal MRI features, that uncover disease subtypes in TLE not visible through conventional analysis. In one study, classifiers trained on patient-specific disease factor compositions predicted response to anti-seizure medications with 76% accuracy and surgical outcome with 88% accuracy, suggesting that dimensional disease characterization may offer superior prognostication compared to traditional categorical approaches.

### 7.4 AI-enhanced multimodal data fusion

A key frontier is the development of AI systems that integrate data across multiple imaging modalities rather than analyzing each in isolation.

**Combined MRI and PET**. Several studies have demonstrated that combining structural MRI features with FDG-PET metabolic data through ML frameworks improves FCD detection beyond either modality alone. Surface-based multimodal profiling that integrates cortical thickness, FLAIR intensity, and PET uptake values can enhance both sensitivity and specificity of lesion detection, particularly for subtle type II lesions.

**Early surgical referral**. (Wissel et al. 2023, 2024) developed ML-based automated alert systems that identified epilepsy surgery candidates early in their clinical trajectory. In a randomized controlled trial published in *Epilepsia* (2023), automated ML alerts increased epilepsy surgery referrals, and a subsequent prospective validation study in *Neurology* (2024) confirmed that ML models could identify surgery candidates with high accuracy using routine clinical data.

Multimodal decision support systems. Li et al. (2023) demonstrated the feasibility of using hybrid PET/ MRI to guide SEEG electrode placement and subsequent radiofrequency thermocoagulation in MRI-negative epilepsy, illustrating how multimodal imaging can be directly translated into interventional therapeutic workflows. While individual studies report promising accuracy for ML-based outcome prediction, significant translational gaps remain—including the absence of prospective multicenter validation, limited external generalizability, and lack of integration into clinical workflows (see Table 5).

**Table 5:** Key AI/ML studies in multimodal epilepsy neuroimaging.

Study	AI/ML method	Input data	Application	Key results
Gill et al. (2021) (^*^Neurology^*^)	Deep CNN with Bayesian uncertainty	3D T1w + FLAIR MRI; 148 patients, 9 centers	FCD detection	Sensitivity 93% overall, 85% MRI-negative; specificity 89%; 6 FP/patient
Sultana et al. (2021) (^*^Brain^*^)	Neural network on surface features	T1w ± FLAIR; 1,015 subjects, 22 centers	FCD detection (MELD)	Sensitivity 85% gold-standard subcohort, 67% full cohort; interpretable reports
Ripart et al. (2025) (^*^JAMA Neurol^*^)	Graph neural network	Surface features; 703 patients, 23 centers	FCD detection (MELD Graph)	Sensitivity 81.6% seizure-free, 63.7% MRI-negative; PPV 67%−76%
Kersting et al. (2025) (^*^Epilepsia^*^)	6 models compared (MAP18, MELD, deepFCD, nnUNet variants)	T1w + FLAIR; 329 subjects, 4 centers	FCD detection comparison	3D-nnUNet best balanced (F1 = 0.58, specificity 86%); deepFCD highest detection (82%) but 0% specificity
Dashtkoohi et al. (2025)	Meta-analysis of 41 studies	MRI (various)	FCD detection	Pooled sensitivity 0.81, specificity 0.92
Memarian et al. (2015)	LS-SVM with mRMR feature selection	iEEG + MRI + clinical data	MTLE outcome prediction	95% accuracy; multimodal superior to unimodal
Munsell et al. (2015)	Elastic net + SVM	DTI structural connectome	TLE outcome prediction	80% TLE vs control accuracy; 70% outcome prediction
Sheikh et al. (2024) (^*^Sci Rep^*^)	ML classifiers	Peri-ictal scalp EEG; 294 patients	Temporal lobe resection outcome	AUC 0.98; accuracy >90%
Wang et al. (2023) (^*^Sci Transl Med^*^)	Bayesian inference + neural mass model	T1w + DWI + SEEG; 53 patients	VEP: EZN estimation + virtual surgery	Precision 0.6; FDR 0.028 (seizure-free) vs. 0.407 (non-seizure-free)
Corona et al. (2023) (^*^Brain^*^)	Graph centrality + virtual implantation	HD-EEG + MEG; 37 patients	Non-invasive network mapping	PPV/NPV ≥55%; outperformed dipoles; valid on spike-free data

### 7.5 Challenges and barriers to clinical translation

Despite the impressive performance metrics reported in research settings, several critical barriers impede the clinical translation of AI in epilepsy neuroimaging.

**Generalizability**. Most AI algorithms are trained and validated on retrospective data from tertiary epilepsy centers, introducing selection bias toward more severe or atypical cases. Performance often degrades when algorithms are applied to data from centers, scanners, or patient populations not represented in training. The MELD project's multicenter approach addresses this partially, but its performance variation across centers (detection rates 46%−54%) highlights the ongoing challenge. The observation that no single algorithm achieves universally optimal performance underscores the need for ensemble approaches or center-specific calibration.

False positive burden. Even the best-performing algorithms produce substantial numbers of false positives (typically 5–6 per patient for deep learning FCD detectors), which require expert review and can lead to unnecessary invasive investigations. Reducing false positives while maintaining sensitivity remains a critical engineering challenge. From a clinical standpoint this false-positive burden is decisive: a positive AI detection cannot by itself justify stereo-EEG implantation or resection, and must be corroborated by concordant structural, metabolic, or electrophysiological evidence before it can influence a surgical decision. A high reported sensitivity is therefore clinically useful only to the extent that its detections can be trusted and independently validated.

**Interpretability**. Clinical adoption requires that AI predictions be interpretable and trustworthy. The MELD project's emphasis on generating feature-level saliency maps and patient-specific reports represents a model for clinical integration, but many deep learning algorithms remain “black boxes” whose predictions are difficult for clinicians to evaluate critically.

**Prospective validation**. The majority of published studies are retrospective. The absence of large-scale prospective trials demonstrating that AI-guided decisions improve patient outcomes—not just diagnostic accuracy—remains the most significant barrier to clinical adoption. The EPINOV trial for VEP and emerging prospective studies for FCD detection algorithms represent important steps toward filling this evidence gap.

**Regulatory and infrastructure barriers**. Clinical deployment of AI tools requires regulatory approval, integration with existing PACS/clinical workflows, computational infrastructure, and ongoing quality assurance. Few epilepsy-specific AI tools have achieved the regulatory clearance and clinical integration necessary for routine use.

**Methodological pitfalls in model development**. Beyond generalizability, several issues specific to machine learning warrant caution when appraising reported performance. Class imbalance—epileptogenic voxels or lesional cases are vastly outnumbered by normal ones—can inflate accuracy metrics unless explicitly addressed. Data leakage, in which information from the test set influences training through subject overlap, feature selection performed before cross-validation splitting, or normalization across the whole dataset, can produce optimistic estimates that fail to generalize. Most importantly for epilepsy surgery, many outcome-prediction models are trained to reproduce the resection zone rather than the true epileptogenic zone; because the resection zone is itself an imperfect surrogate, such models risk learning surgical decision patterns rather than epileptogenicity, and a model that predicts what was resected is not equivalent to one that predicts where seizures arise. Finally, the epileptogenic zone can only be inferred retrospectively from seizure freedom after complete resection, so the very target these algorithms are trained against is uncertain—an intrinsic ceiling on achievable and verifiable performance.

### 7.6 Summary

AI and ML methods have demonstrated substantial potential across the entire presurgical evaluation pipeline: detecting subtle epileptogenic lesions invisible to human readers, extracting quantitative features from multimodal imaging, predicting surgical outcomes, and simulating the effects of surgical interventions through personalized brain models. The field has progressed from proof-of-concept studies to multicenter-validated, open-access tools (MELD, deepFCD) and prospective clinical trials (EPINOV). However, the translation from research performance to clinical impact requires addressing persistent challenges in generalizability, false positive reduction, interpretability, and prospective validation. The integration of AI with multimodal neuroimaging—combining the complementary information from structural MRI, PET, MEG, and electrophysiology within unified computational frameworks—represents the most promising direction for achieving the paradigm-shifting improvements in presurgical evaluation that the field urgently needs.

***Clinical bottom line:*** *across all four domains, AI in multimodal epilepsy imaging remains predominantly at the proof-of-concept stage. Automated FCD detection is the most mature application but is constrained by false positives and by performance that falls outside its training distribution, while outcome-prediction and multimodal-fusion models are promising yet almost entirely retrospective. No epilepsy-specific AI tool has yet demonstrated in a prospective trial that its use improves patient outcomes rather than diagnostic accuracy alone, and these methods should currently be regarded as decision-support adjuncts under expert supervision rather than as validated, deployable clinical tools. Maturity: research and early translational*.

## 8 Challenges and future directions

### 8.1 Methodological heterogeneity and standardization

A persistent barrier to the clinical adoption of multimodal neuroimaging in epilepsy is the absence of standardized protocols for data acquisition, processing, and integration. Each imaging modality involves a complex pipeline—from acquisition parameters to preprocessing, feature extraction, and statistical analysis—and methodological choices at each stage can substantially influence results. For structural MRI, the ILAE Neuroimaging Task Force has established the HARNESS-MRI protocol, recommending core sequences (3D T1-weighted with isotropic voxel size ≤ 1 mm, 3D FLAIR, high-resolution hippocampal T2) to ensure consistent high-quality acquisitions for presurgical evaluation. However, comparable standardization efforts for other modalities and for multimodal integration workflows remain nascent.

In network neuroscience, as discussed in Section 5, the lack of consensus on parcellation schemes, connectivity metrics, frequency bands, thresholds, and graph-theoretic measures renders cross-study comparisons unreliable. Two studies analyzing the same patient cohort with different methodological choices may reach divergent conclusions about network topology and its relationship to surgical outcome. Similarly, AI/ML algorithms for lesion detection employ diverse architectures (surface-based vs. voxel-based, 2D vs. 3D, CNN vs. GNN), training strategies, and evaluation metrics, complicating efforts to identify which approaches offer genuine clinical advantages. The head-to-head comparison by Kersting et al. (2025) revealed substantial performance variation across six FCD detection algorithms on the same multicenter dataset, underscoring the urgency of standardized benchmarking.

Future progress requires coordinated international efforts to establish consensus protocols encompassing acquisition parameters for each modality, standardized preprocessing pipelines, unified evaluation frameworks, and open-access benchmark datasets. Initiatives such as the MELD project, which made its code, protocols, and data openly available across 22 centers, provide a template for this collaborative approach.

### 8.2 The MRI-negative challenge

Despite advances in imaging technology, a substantial proportion of DRE patients—estimated at 20%−40% in contemporary surgical series—remain MRI-negative on conventional 3T imaging. This group consistently demonstrates inferior surgical outcomes compared to lesional cases, yet paradoxically may derive the greatest benefit from multimodal evaluation approaches.

Ultra-high-field 7T MRI represents a promising frontier for addressing this challenge. A systematic review by van Lanen et al. (2021) pooling 16 studies including 275 patients found that 7T MRI provided a diagnostic gain ranging from 8 to 67% across individual studies, with a pooled extra yield of 31% over conventional field strengths. Focal cortical dysplasia (54%), hippocampal sclerosis (12%), and gliosis (8.1%) were the most frequently identified histopathological entities. Wang et al. (2020) demonstrated in a cohort of 67 patients with non-lesional 3T MRI that unaided 7T visual review detected subtle lesions in 22% of cases, and when aided by morphometric analysis program (MAP) post-processing, the total yield increased to 43%. Critically, 7T-identified lesion locations were concordant with intracranial EEG ictal onset in 81% (13/16) of cases, and complete resection of 7T-identified lesions was significantly associated with seizure freedom (p = 0.03). However, the 7T Epilepsy Task Force consensus protocol implementation study by Hangel et al. (2024) reported a more modest single-center diagnostic gain of 19%, and noted that B1+ inhomogeneity and subject motion remain significant limitations at ultra-high field. More broadly, the practical constraints of 7T imaging temper its role: scanners remain scarce and geographically concentrated, motion and susceptibility artifacts and specific-absorption-rate limits complicate acquisition, and the incremental yield over an optimized 3T HARNESS protocol is still uncertain, so that 7T is at present best reserved for selected MRI-negative cases evaluated at specialized centers rather than adopted as a routine first-line sequence.

Beyond 7T, multimodal integration itself serves as a strategy for the MRI-negative population. The convergence of abnormalities identified by FDG-PET hypometabolism, MEG spike localization, ictal SPECT hyperperfusion, and advanced MRI post-processing can collectively delineate a region concordant with the presumed epileptogenic zone even when no single modality identifies a clear lesion. The combination of AI-based lesion detection algorithms (achieving 63%−85% sensitivity in MRI-negative FCD) with multimodal concordance analysis represents the most promising near-term approach for this challenging population.

### 8.3 Validation deficits: from retrospective to prospective evidence

The evidence base for multimodal neuroimaging in epilepsy is overwhelmingly retrospective. Most studies suffer from selection bias (evaluating only patients who ultimately underwent surgery), small sample sizes (typically 20–60 patients), single-center designs, and the absence of standardized outcome measures. These limitations render reported diagnostic accuracies and outcome predictions optimistic and of uncertain generalizability.

The transition to prospective, multicenter validation represents the most critical need in the field. Only a handful of ongoing prospective trials directly test whether multimodal approaches improve clinical outcomes. The EPINOV trial (NCT03643016) for the Virtual Epileptic Patient framework, discussed in Section 5, represents the largest such effort, with an expected enrollment of 356 patients and an evaluation horizon extending through 2025. For AI-based lesion detection, no completed prospective trial has yet demonstrated that algorithm-guided decisions lead to better surgical outcomes than standard clinical practice.

Future validation studies must be designed to answer the pragmatic clinical question: does the addition of multimodal imaging and AI-guided analysis to standard presurgical evaluation improve the proportion of patients who achieve seizure freedom? Answering this question requires randomized or registry-based comparative effectiveness studies with adequate follow-up, standardized outcome measures (ILAE classification), and pre-specified analysis plans.

### 8.4 Translational and infrastructure barriers

Even when multimodal approaches demonstrate scientific validity, their clinical implementation faces substantial practical barriers.

Access and resource disparities. Advanced imaging modalities—PET, MEG, 7T MRI, SPECT—require expensive equipment, specialized technical personnel, and dedicated infrastructure that are available only at large academic epilepsy centers. This creates a significant equity gap: patients without timely access to a specialized epilepsy center may not have access to the modalities that could most benefit their evaluation. The development of AI tools that can extract maximum information from widely available modalities (particularly 3T MRI) partially addresses this concern, but the most effective multimodal approaches inherently require multiple imaging technologies.

**Workflow integration**. Multimodal data integration is computationally demanding and typically requires specialized expertise in image co-registration, fusion, and interpretation. Few epilepsy centers have established clinical workflows that routinely incorporate quantitative multimodal analysis into surgical planning conferences. Standardized software platforms (such as SWANE—Standardized Workflow for Advanced Neuroimaging in Epilepsy) are emerging, but their adoption remains limited.

**Multidisciplinary expertise**. The interpretation of multimodal neuroimaging data in the context of seizure semiology, neuropsychological profiles, and electrophysiological findings requires multidisciplinary teams with expertise spanning neuroradiology, nuclear medicine, neurophysiology, computational neuroscience, and epilepsy surgery. Training pipelines for this type of cross-disciplinary expertise are underdeveloped.

**Computational requirements**. Personalized brain modeling approaches such as VEP require high-performance computing infrastructure, specialized software, and substantial computational time. While these tools are scientifically promising, their deployment in routine clinical settings remains challenging without significant simplification and automation.

### 8.5 Emerging technologies and future paradigms

Several emerging technologies and conceptual advances are poised to reshape multimodal neuroimaging in epilepsy.

**Simultaneous multimodal acquisitions**. Integrated PET/MRI systems enable simultaneous acquisition of metabolic and structural/functional data, eliminating co-registration errors and reducing patient burden. The combination of FDG-PET metabolic mapping with simultaneous fMRI functional connectivity analysis within a single scanning session offers unprecedented opportunities for multimodal integration. Similarly, EEG-fMRI co-registration during simultaneous recordings enables the mapping of hemodynamic correlates of interictal epileptiform discharges with millisecond-to-millimeter precision.

**Advanced diffusion imaging**. Beyond conventional DTI, techniques such as high angular resolution diffusion imaging (HARDI), diffusion spectrum imaging (DSI), neurite orientation dispersion and density imaging (NODDI), and fixel-based analysis provide more nuanced characterization of white matter microstructure that may improve the sensitivity of structural connectivity mapping in epileptic networks.

**Deep learning for end-to-end multimodal fusion**. Current AI approaches largely analyze each modality independently before combining results. Future architectures employing attention mechanisms, graph transformers, and multimodal representation learning may achieve true end-to-end fusion, learning the optimal strategy for combining information across modalities without requiring explicit feature engineering.

**Digital twins and virtual surgery**. Building on the VEP framework, the concept of patient-specific digital brain twins—continuously updated computational models that incorporate all available multimodal data—could enable virtual testing of multiple surgical strategies before committing to an intervention. Virtual resection, disconnection, and neuromodulation simulations could compare predicted seizure outcomes across treatment options, moving epilepsy surgery from an empirical to a model-guided paradigm.

Neuromodulation. For patients who are not surgical candidates or who have failed resection, neuromodulation—including vagus nerve stimulation, deep brain stimulation of the anterior thalamic nucleus, and responsive neurostimulation—guided by multimodal imaging-derived biomarkers represents an emerging therapeutic frontier. Network-based targeting of neurostimulation, informed by patient-specific connectome analysis, could optimize electrode placement and stimulation parameters for maximum seizure control.

### 8.6 Toward precision epilepsy surgery

The ultimate vision emerging from the convergence of multimodal neuroimaging, network neuroscience, AI, and computational modeling is that of precision epilepsy surgery. In this paradigm, each patient's presurgical evaluation would proceed through a structured pipeline: high-resolution multimodal imaging generates comprehensive data on brain structure, metabolism, connectivity, and electrophysiology; AI algorithms automatically extract quantitative features and detect subtle lesions; network analysis identifies the patient-specific epileptogenic network topology; computational models simulate the expected outcomes of different surgical interventions; and the surgical team selects the intervention predicted to maximize seizure freedom while minimizing functional deficits.

Achieving this vision requires addressing the methodological, validation, and translational barriers outlined above. Most critically, it requires a paradigm shift from viewing multimodal neuroimaging as a collection of independent tests to treating it as an integrated computational pipeline where each modality contributes a complementary piece of information to a unified patient model. The field is at an inflection point where the scientific foundations for this integrated approach have been established, but the clinical evidence, standardized workflows, and practical infrastructure needed for routine implementation remain works in progress.

Two clarifications temper this vision. First, “precision epilepsy surgery” should be understood operationally rather than as a slogan: we use it to denote the tailoring of the diagnostic work-up and the surgical intervention to the individual patient's anatomy, epileptic network, and functional organization, guided by quantitatively integrated multimodal data, rather than any specific technology. Second, it is important to recognize that contemporary presurgical practice is already individualized—each case receives a bespoke diagnostic pathway, and multimodal information is already synthesized within the multidisciplinary epilepsy surgery conference. The advance offered by the methods reviewed here is therefore not the introduction of individualization *per se*, but the prospect of making that integration more quantitative, reproducible, and explicit; multimodal imaging supports, and does not replace, expert multidisciplinary judgment.

## 9 Conclusion

Drug-resistant epilepsy remains one of the most challenging conditions in clinical neuroscience, affecting approximately one-third of individuals with epilepsy and carrying profound consequences for quality of life, cognition, and mortality. Epilepsy surgery offers the most effective treatment, yet its success depends critically on the accurate localization of the epileptogenic zone—a task that no single imaging modality or clinical test can reliably accomplish in isolation.

This review has examined the current evidence for multimodal neuroimaging integration in the presurgical evaluation of DRE, spanning individual imaging modalities, technical fusion approaches, clinical evidence for specific multimodal combinations, network-based analyses, and AI/ML applications. Several key conclusions emerge from this synthesis.

seizure freedom rates of 70%−90% in favorable, predominantly lesional and temporal-lobe series, compared to 30%−50% when modalities are discordantFirst, multimodal concordance consistently predicts superior surgical outcomes. Studies across diverse patient populations demonstrate that agreement between two or more imaging modalities in localizing a candidate epileptogenic region is associated with or when only a single modality is informative. This concordance principle applies across modality combinations—structural MRI with FDG-PET, MEG with MRI, ictal SPECT with structural imaging—and provides the strongest current rationale for comprehensive multimodal evaluation.

Second, the paradigm shift from discrete epileptic focus to epileptic network has fundamentally reframed the goals of presurgical imaging. Graph-theoretic and computational modeling approaches, exemplified by the Virtual Epileptic Patient framework, demonstrate that surgical success depends not only on identifying the seizure onset zone but on characterizing and disrupting the broader network that sustains epileptogenic activity. Multimodal imaging is uniquely positioned to capture this network complexity, with each modality providing a complementary window onto the structural, functional, and metabolic dimensions of the epileptic network.

Third, AI and machine learning have achieved clinically meaningful performance in specific applications, particularly automated FCD detection (pooled sensitivity 81%, specificity 92%) and surgical outcome prediction. However, the gap between research performance and clinical implementation remains wide. Prospective multicenter validation, regulatory approval, and workflow integration are prerequisite steps that the field must prioritize.

Fourth, the MRI-negative population—comprising 20%−40% of surgical candidates—represents both the greatest clinical challenge and the population most likely to benefit from multimodal approaches. The combination of ultra-high-field MRI, AI-based lesion detection, multimodal concordance analysis, and network-based computational modeling offers a converging set of tools that can collectively address this unmet need.

Looking forward, the trajectory of the field points toward an integrated computational framework—combining multimodal imaging data acquisition, AI-driven feature extraction and lesion detection, patient-specific network modeling, and virtual surgical simulation—that would transform presurgical evaluation from a qualitative, expert-dependent process into a quantitative, model-guided pipeline. While significant scientific, methodological, and translational barriers remain, the foundational elements for this transformation are now in place. The challenge for the coming decade is to bridge the gap between scientific demonstration and clinical impact through large-scale prospective validation, standardized protocols, accessible computational tools, and equitable access to advanced imaging technologies.

Ultimately, the goal is to ensure that every patient with drug-resistant epilepsy benefits from the full potential of modern neuroimaging science—enabling more patients to be identified as surgical candidates, more epileptogenic zones to be accurately localized, and more individuals to achieve the life-changing outcome of seizure freedom.
